# Cohort profile: Studies of Work Environment and Disease Epidemiology-Infections (SWEDE-I), a prospective cohort on employed adults in Sweden

**DOI:** 10.1371/journal.pone.0217012

**Published:** 2019-05-15

**Authors:** Francesca Ghilotti, Anneli Julander, Per Gustavsson, Annika Linde, Olof Nyrén, Amelie Plymoth

**Affiliations:** 1 Department of Medicine, Clinical Epidemiology Unit, Karolinska Institutet, Stockholm, Stockholm, Sweden; 2 Department of Statistics and Quantitative Methods, University of Milano-Bicocca, Milan, Italy; 3 Institute of Environmental Medicine, Karolinska Institutet, Stockholm, Sweden; 4 Department of Medical Epidemiology and Biostatistics, Karolinska Institutet, Stockholm, Sweden; Sciensano, BELGIUM

## Abstract

The aim of this article is to provide a detailed description of the SWEDE-I cohort, a prospective study designed to investigate work-related risk factors for transmission of viral infections. A total of 2,237 subjects aged 25–64, working and residing in Eskilstuna (central Sweden), enrolled in the study in August 2011. They filled in five detailed questionnaires including information on demography, personal characteristics, work tasks, work place, contact patterns, family structure, health status, physical activity and diet. During a 9-month follow-up period, the participants self-reported—via internet or telephone—any onset of fever, upper respiratory tract infection, or gastroenteritis immediately as they occurred. For each disease episode, the participants were asked to submit a self-sampled nasal swab for viral diagnosis. In total, 1,733 disease reports were recorded and 1,843 nasal swabs were received, of which 48% tested positive for one or more of 14 analyzed viruses. The cohort has been used to date to study diet, sleep and physical activity as determinants for upper respiratory tract infections. Analyses of contact patterns and occupational circumstances as risk factors for the transmission of infections are ongoing. The SWEDE-I study should be seen as a first pioneering effort to provide new insight in the epidemiology and prevention of viral infections. Potential joint collaborations can be discussed with the principal investigators.

## Introduction

Acute viral infections of the upper respiratory and gastrointestinal tracts constitute a large part of the total short-term disease burden among adults of productive age. These aliments explain a significant part of the disease-related loss of production. It was previously found that respiratory disorders and gastroenteritis accounted for 50–60% of all episodes of absence from work [1]. Already in the early 2000s, the annual economic cost of lost productivity due to the common cold in the USA was estimated to approach $25 billion USD, [2] while others estimated the total cost at $40 billion USD [3].

A more recent Swedish study showed that the proportion of workers who reported at least one day a year of absenteeism due to rhinitis and common cold was 37%, whereas 72% reported to have worked while sick with subsequent reductions in productivity [4]. The total annual costs for productivity loss in Sweden were estimated at €2.7 billion a year [4].

Despite these impressive figures, surprisingly little has been done to study risk factors for transmission of acute respiratory infections and viral gastroenteritis and to explore alternative possibilities for prevention of work-related infectious transmission. With few exceptions [5, 6] current public counseling rests heavily on family and community studies from the 1950’s and 70’s [7] and viral-challenge studies in laboratory settings done in the 1990s [8].

In these studies, data about work-related factors that can facilitate or hinder spread and clinical manifestation of infections were scarce and patchy. Hand washing, psychological stress, social support, and shift work may modify the risk of viral respiratory infection [8–11]. However, data on the preventive role of physical interventions, e.g. hand washing, in adult populations are rather weak and the results are conflicting [12]. The role of physical activity is uncertain [13, 14]. Body cooling seems to be unrelated to risk [15], but sudden changes of temperature at work were significantly associated with the risk of community-acquired pneumonia in one study [16]. Sleep duration and efficiency was shown to be strongly linked to susceptibility to experimental inoculation of rhinovirus, adding support to the importance of shift work as a facilitating factor [17, 18]. However, to our knowledge, the effects of long sleep duration on infections are not well established. Smoking, and to a lesser extent passive smoking, appears to be important [19, 20], and the importance of dust, particles and airborne irritants needs to be investigated.

The general population cohort study SWEDE-I (Studies of Work Environment and Disease Epidemiology-Infections) was conceived to fill some of the knowledge gaps. The specific purposes of the cohort were to: (i) determine work-related factors relevant to the spread of common acute respiratory infections and viral gastroenteritis, both overall and by causative viral agent; (ii) provide empirical data on factors that affect the probability of transmission of common viral infections in various work-related settings, in order to improve the epidemic models needed for predictions and planning when major outbreaks are foreseen. After a pilot study in the spring of 2011, the SWEDE-I study was carried out between August 22, 2011 and May 31, 2012.

The aim of this cohort profile paper is to provide a comprehensive description of the SWEDE-I cohort as a research resource for potential collaborations, including an overview of the collected data, a description of the baseline characteristics and a summary of the main results published so far.

## Cohort description

### Study design and study population

**Fig 1:**
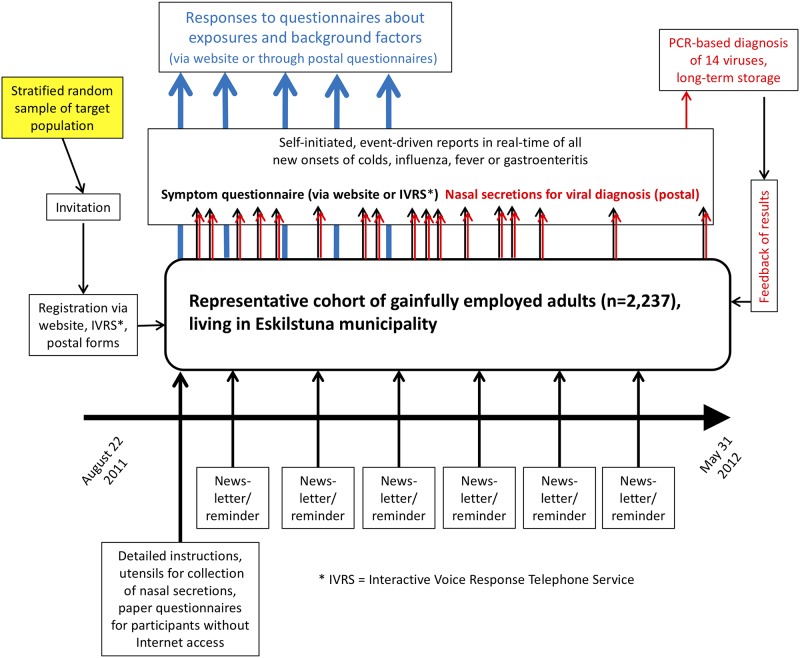
Overview of the study design, including the infectious disease surveillance scheme.

Eskilstuna, an industrial town in central Sweden with around 98,000 inhabitants, was chosen as the study site due to its circumscribed and stable population who both reside and work in the community, and for its variety of manufacturing industries. Inclusion criteria were, besides living in Eskilstuna, being gainfully employed and aged 25–64 years. A representative sample of this particular target population was created by Statistics Sweden through record-linkage between its Labour Market Register and the Total Population Register. To achieve a premeditated sample size of 2,200, based on the low observed participation rates in earlier population-based infectious disease surveillance studies with a similar commitment [21], postal invitations were sent to an age- and sex-stratified random sample of 14,008 individuals in August 2011. In particular, Bexelius et al. [21] found a moderate under-representation of men, people with low education, and people representing one-person households, and a more marked under-representation of the age stratum 18–39 years. For this reason, men and subjects aged 25–44 years were over-sampled in our study. Fig 1 shows an overview of the study design, including the infectious disease surveillance scheme.

**Fig 2:**
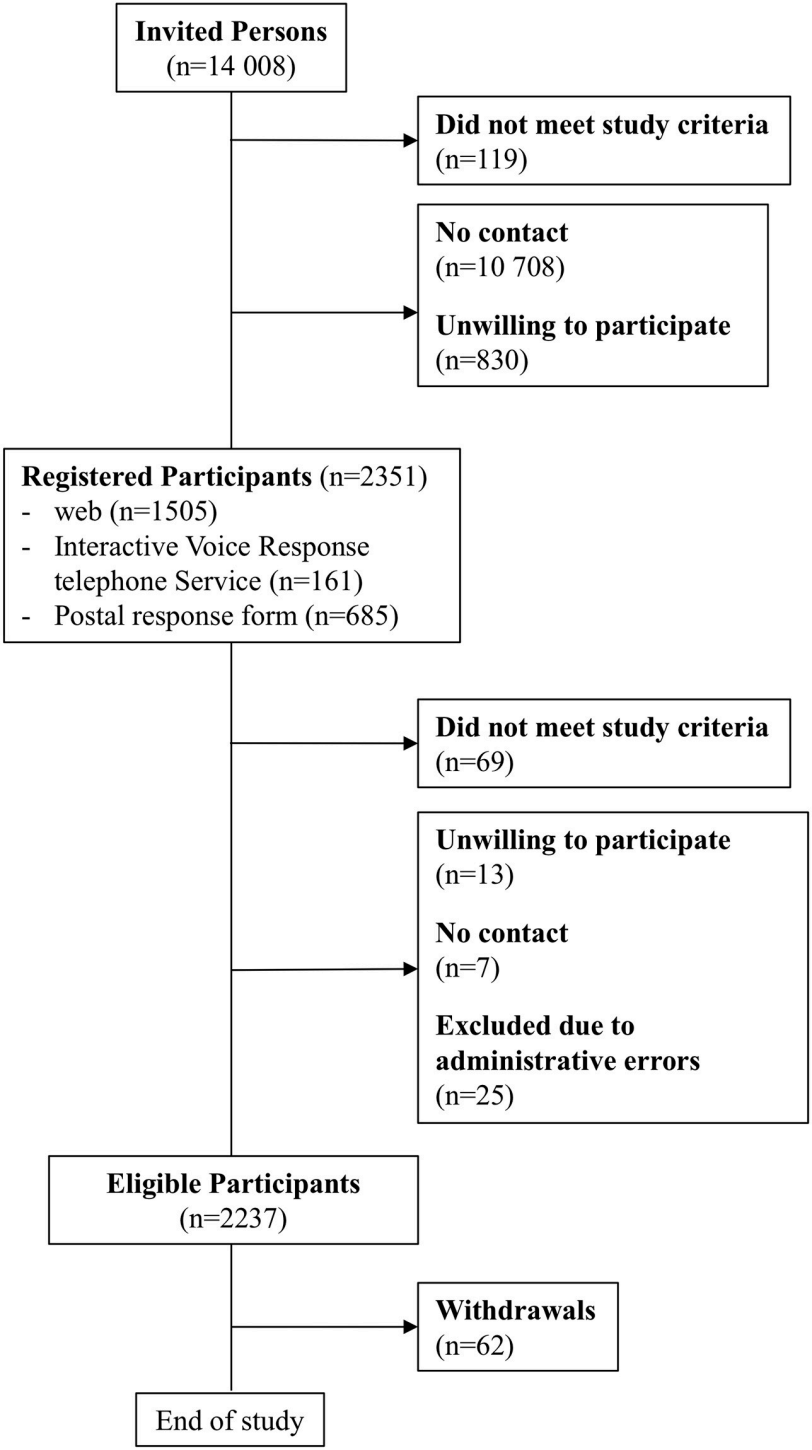
Study flow diagram of recruitment, enrolment and follow-up.

The invitees registered themselves as participants on the study’s website, via a specially designed interactive voice response telephone service (IVRS), or by sending in a postal response form. Individuals who stated, in response to the invitation (n = 119) or the first questionnaire (n = 69), that they were not currently working (unemployed, retired, students, on long-term sick or parental leave) were excluded due to non-eligibility. After one reminder, the final cohort comprised 2,237 participants. Fig 2 shows the study flow diagram of recruitment, enrolment and follow-up.

The study protocol was approved by the Regional Ethics Review Board in Stockholm, Sweden (dnr 2011/360-31/2). All participants gave their written informed consent.

### Assessment of exposures

Participants completed five questionnaires (Table 1) covering a wide range of suspected determinants for exposure to and transmission of common viral infections, as well as factors tentatively linked to individual susceptibility and propensity for reporting. The English version of the questionnaires is included as supplementary material (S1–S5 Files). The first four were offered in both formats, either web or paper. Data on dietary intake and physical activity were assessed in the fifth questionnaire, only available on the web; for this purpose the two validated interactive questionnaires, Active-Q and MiniMeal-Q were used [22, 23]. All questionnaires were available immediately after entering into the study for subjects choosing to respond on the web; for subjects who preferred the paper version, the first four questionnaires were sent out monthly one-by-one over the first half of the follow-up period. After enrolment, an experienced occupational hygienist classified the occupation using the Swedish Standard Classification of Occupations from 1996 (SSYK 96 on 4-digit level, corresponding to the International Standard Classifications of Occupations 1988) and classified the industry using the Swedish Standard for Industrial Classification (SNI 92 on 3-digit level). An occupational classification is based on jobs and tasks performed whereas an industrial classification is based on economic sector of the company [24].

**Table 1:** Description of the 5 questionnaires sent to the 2,237 participants enrolled in the cohort and their response rate.

Questionnaire	Title	Number of Variables	Number of questionnaire received	Response rate (%)
Q1	Your work and work place	88	2,033	91
Q2	Specific conditions at work	65	1,900	85
Q3	About you, your family and your contacts	52	1,815	81
Q4	About your health status	67	1,810	81
Q5	Physical activity and dietary habits	392	1,544	69

### Follow-up and outcome assessment

The participants were instructed to self-report, on their own initiative, all onsets of fever, upper respiratory tract infection, and gastroenteritis immediately as they occurred during the entire study period (August 22, 2011 until May 31, 2012). Frequent reminders, together with monthly newsletters encouraged high compliance. The newsletters contained feedback about observed trends in occurrence of the investigated viruses, information about the study and the tasks to be carried out by the participants, along with plenty of “infotainment” related to infectious diseases. Passive follow-up of the kind practiced in the present study, relying on self-initiated, event-driven infectious disease reporting, has been thoroughly evaluated from various aspects, including validity and completeness of reporting [21, 25]. Despite substantial under-reporting, this seems to be remarkably constant over time so that adjustments using a constant correction factor would potentially restore validity of incidence rates. In additions, epidemic curves derived from self-reporting correlate well with curves obtained from the sentinel surveillance curves [21].

#### Symptoms

All onsets of fever, upper respiratory tract infection, and gastroenteritis were self-reported. The participants could alternate between a website and an automated IVRS as the channel for their reports. Irrespective of the contact mode, the sick report included 21 responses to a tree-structured symptom questionnaire. It comprised symptoms known to be associated with acute respiratory infections or gastroenteritis: sore throat, coughing, runny nose, headache, fever and body ache. Information about exact date of onset, contacts with people in the family, in the workplace, or elsewhere who had similar symptoms before onset, and consultation with a doctor after onset were also collected. The symptom questionnaire is included in the supplementary material (S6 File). Based on pre-defined algorithms, the diseases could be classified as influenza-like illness (ILI), [26] common cold, [27] gastroenteritis, or other/unclassifiable.

#### Viral agents

Concurrent with every symptom report (ILI, common cold, gastroenteritis, or other/unclassifiable), the participants were instructed to take a sample of secretions from the nose. Two kits with regular nylon flocked dry swabs in plastic tubes (Nordic Biolabs, Täby, Sweden), along with thorough instructions, had been sent to each participant shortly after entry into the cohort. The used kits were returned via regular pre-paid mail. When the last kit had been returned, the participant was automatically supplied with a new one. Viral diagnosis was performed by the Clinical Microbiology Laboratory at Karolinska University Hospital, Solna, using real-time polymerase chain reaction (PCR) systems in 384-well plates [28]. The samples were analyzed for 14 different viruses: Rhinovirus; respiratory syncytial virus (RSV); influenza A, A(H1N1) and B; parainfluenza 1, 2 and 3; human coronavirus (hCOV) 229E, HKU1, NL63 and OC43; enterovirus; and metapneumovirus (MPV). Remaining material was stored at—70°C for possible future supplementary analyses. Each participant received a unique code which, combined with the individual’s unique national registration number, gave access to a secure website with the viral test results. The feasibility of self-sampling providing viable samples for molecular detection has previously been demonstrated [29].

#### Attrition during follow-up

To preserve validity, participants were exhorted to actively de-register from the study if they found participation too burdensome, rather than to remain in the study without factual participation. Of the 2,237 participants in the cohort; 62 quit the study before its end due to e.g. emigration and unwillingness to continue. Data on those participants is included until their drop-out date.

## Findings to date

Demographic and clinical characteristics of the study cohort are shown in Table 2. The cohort consists mainly of daytime workers (85%) and the majority of individuals reported a good or very good health status (81%). Compared with the working age Swedish population in 2011, the cohort members were on average older, more educated, and with a higher predominance of female gender. Contrarily, the proportion of daily smokers (age-class 45–64 years), of obese individuals and of individuals with a good health status (age-class 30–44 years) was similar to the ones in the target population. Detailed results on the comparison are presented as supplementary material (S1 Table); data on the general Swedish population were derived from Statistics Sweden and Folkhälsomyndigheten, the Public Health Agency of Sweden [30, 31].

**Table 2:** Demographic and clinical characteristics of the 2,237 participants enrolled in the cohort for the study of work-related risk factors for transmission of viral infections (SWEDE-I), Sweden, August 2011–May 2012.

	n/N^a^	%
Sex		
Men	918/2,237	41
Age group in years		
25–34	284/2,237	13
35–44	718/2,237	32
45–54	592/2,237	26
55–64	643/2,237	29
Body Mass Index		
< 18.5	10/1,734	1
18.5–24.9	817/1,734	47
25–29.9	651/1,734	37
≥ 30	256/1,734	15
Highest attained education		
Secondary school (≤ 9 years)	160/1,797	9
Upper secondary school (11–13 years)	596/1,797	33
University/college < 3 years	260/1,797	15
University/college ≥ 3 years	596/1,797	33
Other post-secondary education	185/1,797	10
Smoking status		
Current smokers	203/ 1,812	11
Working hours		
Daytime	1,709/2,018	85
Evenings/nights	74/2,018	4
Shift work	152/2,018	7
Other	83/2,018	4
Healthcare work		
Yes	349/1,898	18
Household size		
1	251/1,814	14
2	704/1,814	39
3	326/1,814	18
4	396/1,814	22
≥ 5	137/1,814	7
Household income		
< 300,000 SEK	173/1,808	10
300,000–499,999 SEK	499/1,808	28
500,000–799,999 SEK	873/1,808	48
≥ 800,000 SEK	263/1,808	14
Health status		
Good/Very good	1,457/1,801	81
Neither good nor poor	297/1,801	16
Poor/Very poor	47/1,801	3
Number of disease reports sent		
0	1,158/2,237	52
1	654/2,237	29
2	267/2,237	12
≥ 3	158/2,237	7
Number of nasal swabs sent		
0	1050/2,237	47
1	729/2,237	32
2	306/2,237	14
≥ 3	152/2,237	7

^a^ The cohort is comprised of 2,237 subjects, but not all responded to all questions asked in the questionnaires.

### Reported infections and PCR-based analyses of 14 viruses

In total, 1,733 disease reports were recorded in the present study and 1,843 nasal swabs were received, of which 48% tested positive for one or more of the analyzed viruses [32]. In total, 1,461 sick reports could be merged with the laboratory data.

It is not surprising that the number of nasal swabs exceeded the number of disease reports. Some individuals, indeed, enjoyed the possibility to have a quick feedback on their test results and to get to know more about the virus description and its associated disease but they did not contribute to the study with their disease reports. This explains part of the 382 swabs that could not be matched, the remaining part is due to timing issues: when the number of days between arrival of the specimen to the lab and disease onset was greater than 15 or smaller than -5 (case where the specimen was sent before the registered onset of disease) laboratory results were not linked to symptoms reported. On the other hand, 272 sick reports could not be merged to nasal swabs. Apart from the same issue of timing, this fact might also be explained by certain symptoms reported. The absence of a nasal sample correlates well with the presence of vomiting and the absence of runny nose.

Further details on specimen results can be found in our Eurosurveillance paper [32]. Briefly, when analyzing the seasonality, we found that the peak in the number of returned swabs was reached in the last week of September 2011, but the proportion of positive tests increased from mid-November until April. Among all samples tested, rhinovirus was the most common virus diagnosed (20.8%), followed by viruses belonging to coronavirus group (16.2%) and influenza (4.8%).

The average number of reported infections and virally diagnosed infections during the 9-month follow-up is reported in Table 3 stratified by the 10 largest occupational sub-major groups and gender. The differences in the number of reported infections were larger among the different occupational sub-major groups than between men and women. The occupational sub-major groups that reported the highest number of infections were those including health care workers (SSYK96 code 32) and preschool (SSYK96 code 33) and primary teachers (SSYK96 code 23) working with younger children. Corporate managers (SSYK96 code 12) reported instead the lowest numbers of infections.

**Table 3:** Descriptive data on the number of women and men in the 10 largest occupational sub-major groups as well as the average amount of reported infections and virally diagnosed infections during the 9-month follow-up.

SSYK96 Code	Occupation	N		Average number of reported infections during 9-month follow-up			Average number or virally diagnosed infections during 9-month follow-up		
		Women	Men	Tot	Women	Men	Tot	Women	Men
12	Corporate managers	59	71	0.6	0.8	0.5	0.2	0.3	0.2
21	Physical, mathematical and engineering science professionals	30	79	0.7	0.8	0.6	0.3	0.2	0.3
23	Teaching professionals	99	34	1	1.1	0.6	0.5	0.5	0.4
24	Other professionals	105	39	0.9	1	0.6	0.4	0.4	0.3
31	Physical and engineering science associate professionals	31	96	0.9	1.2	0.8	0.4	0.5	0.4
32	Life science and health associate professionals	96	11	1	1	1	0.4	0.4	0.5
33	Teaching associate professionals	88	6	1.7	1.7	1	0.6	0.6	0.5
34	Other associate professionals	129	92	0.8	0.9	0.7	0.4	0.4	0.3
41	Office clerks	117	53	0.8	0.9	0.6	0.3	0.4	0.2
51	Personal and protective services workers	246	30	0.8	0.8	0.7	0.4	0.4	0.2

### Diet and upper respiratory tract infections

In one paper, published in 2017, we investigated the relationship between diet and upper respiratory tract infections (URTI) [33]. Using data from the web-based food frequency questionnaire we found an inverse association between intake of vitamin C, vitamin E, docosahexaenoic (DHA) and arachidonic acid (AA) and risk of URTI among women, while intake of vitamin E and zinc was associated with an increased risk of URTI among men [33].

### Physical activity, sleep, and upper respiratory tract infections

In a recently published analysis, we used data from the SWEDE-I cohort to investigate the relationship between sleep, physical activity and occurrence of URTI [34]. The fitted hurdle regression models did not provide support for an association with either physical activity or sleep habits.

## Strengths and limitations

### Strengths

(I) Advantage was taken of a newly developed and validated method for infectious disease surveillance through passive follow-up of a representative cohort, with self-initiated, event-driven infectious disease reporting via automated channels. In addition, the follow-up covered the entire Swedish season of respiratory infections. (II) Viral testing was done on a large scale. Unique information was obtained about the dynamics of viral agent-specific outbreaks/epidemics occurring during nine months in a representative population. Long-term storage of the collected samples allows possible subsequent in-depth molecular studies. (III) Unlike traditional approaches in infectious disease epidemiology, where focus is placed on identifiable encounters with infected individuals, resulting in dichotomous viral exposure assessments, the assessments in this study are probabilistic in nature, based on observed, period-specific incidence rates in the population and self-reported contact patterns. (IV) The extensive set of covariates not only enhances control of confounding and imputation of missing values, but also provides new quantitative information about associations of a large number of occupational and non-occupational factors (e.g. air temperature, dust, hand washing, long sleep, exercise) with risks of common viral infections.

### Limitations

Limitations worth highlighting include uncertain external validity (I) and possible threats to internal validity (II).

(I) First, the study was confined to one medium-sized Swedish industrial town. Second, no more than 16% of the invited individuals participated. Selecting subjects from a restricted source population, however, might enhance feasibility of the study and increase completeness of follow-up [35]. In a small community with high participation density it is also easier to keep participants alerted to their reporting responsibility since information about the study tends to propagate by word-of-mouth. Given the significant commitment required, the low participation rate was expected. In addition, to preserve internal validity, the invitees were discouraged from signing up if they doubted that they were able to completely fulfil the commitments [21]. In the present study, the expected under-representation of men and of the age stratum 25–44 years was compensated for in the recruitment; despite that, the proportion of men and individuals in the age stratum 25–44 is still lower compared to the working age Swedish population in 2011. An older age, a higher degree of education and a predominance of females in respondents compared to the total population have been previously reported also in other epidemiological studies [36, 37] and it might cast doubt on the generalizability. It has been argued, however, that a skillful control for confounding and the understanding of mechanisms of effect [38] might be even more important than representativeness of the sample. Additionally, unintentional non-representativeness might cause greater bias when the probability of participation in the study is affected by the outcome [39], but we have no reason to believe that a common outcome as viral infections is linked to decision to take part in the study. Lastly, the potential lack of representativeness due to self-selection has to be balanced against the potential bias from poor response to follow-up in a more representative sample [39].
(II) Since assessment of exposures was only made once, there may be non-differential misclassification of the more relevant exposures that occurred in the few days that preceded the onset of disease episodes. From a purely biological point of view, this may lead to underestimations of the strength of causal associations, but since aspects of the work environment that are potentially modifiable tend to be more stable over time (e.g. working with young children, working in hospitals, working indoors/outdoors, being in contact with several people…), associations that are of interest from a public health perspective may be less liable to underestimation. Given the prospective nature of the study, exposure misclassification that is differential in regard to the outcome is unlikely. However, since participants did not answer the risk factor questionnaires before the start of follow-up, but rather spread them out over the first months, there is some scope for reverse causation. As all responses are recorded with a time stamp, there are ample possibilities to do sensitivity analyses, where each individual’s start of follow-up is shifted to the point when all the relevant exposure information had been registered. Misclassification of the outcome is another internal validity issue. The total number of reported infections might be inaccurate, in particular we observed that individuals with more missing answers in the exposure questionnaires had a lower number of infections reported. The significant commitment required may have caused in some individuals reporting fatigue which, as a matter of fact, may result in fewer or zero infections being reported. In addition, it is not possible to distinguish between a zero occurrence and a nonresponse, but methods to account for the presence of excess zeros are available [40]. Extensive evaluations of the validity of self-initiated, event-driven infectious disease reports have been done in two previous population-based surveillance cohorts, each followed during one infectious disease season [25]. Briefly, while the specificity was very high (essentially no false positive reporting), there was substantial under-reporting. The estimate was 60%, but since the reference standard measurements (retrospective reporting at the end of the surveillance period) were likely over-estimating the number of disease events due to so called telescoping bias, the true extent of under-reporting is likely to be of the order of 50% [25]. The constancy across sociodemographic strata in the validation studies allays the obvious concern that the under-reporting might be differential in regard to the studied exposures. Some further comfort inheres in the rule of thumb that independent non-differential disease misclassification with perfect specificity will not bias the risk-ratio estimate [41].

## Conclusion

The SWEDE-I study should be seen as a first pioneering effort to lay the foundation for a new avenue of research with the aim of reducing short-term absenteeism due to infections, and to increase preparedness in the event of serious infectious threats.

## Supporting information

S1 File Questionnaire 1. (PDF)

S2 File Questionnaire 2. (PDF)

S3 File Questionnaire 3. (PDF)

S4 File Questionnaire 4. (PDF)

S5 File Questionnaire 5. (PDF)

S6 File Symptom questionnaire. (PDF)

S1 Table Comparison of baseline characteristics between cohort members and the working age Swedish population in 2011. (DOCX)
