# Supplementary material for: Cohort profile: Studies of Work Environment and Disease Epidemiology-Infections (SWEDE-I), a prospective cohort on employed adults in Sweden
Source: PLoS One. 2019 May 15;14(5):e0217012. doi: 10.1371/journal.pone.0217012 (PMC6519895; doi:10.1371/journal.pone.0217012)
Supplement: S1 File — (PDF) [file pone.0217012.s001.pdf]

1. Your work and work place

Jobboch**Smitta** refers only to people who are currently working. **If you are not working** you may unfortunately not join this survey. If this is the case, please just state your current situation below and send in the questionnaire in the self-addressed envelope enclosed. (occ_occupation)

- ☐ On long term sick leave (2)
- ☐ At school, university or similar, including internship (3)
- ☐ On parental leave (4)
- ☐ Work at home (including care of children or relatives, husband/wife) (5)
- ☐ OAP (6)
- ☐ Unemployed (7)
- ☐ Other (8)

Everyone else; please begin with question number 1 below:

1. What has been your main occupation during the last 3 months? (occ_form)

- ☐ Employed, waged labour (1)
- ☐ Self-employed (including farmer) (2)

2. What is your current occupation? *Try to give as detailed a job title as possible (for example: shop assistant in a book store).* (occ_titel)

(text)

____

____

____

3. In total, for how long have you been working in the occupation stated in question number 2? (occ_title_yrs)

- ☐ Less than a year (1)
- ☐ 1-4 years (2)
- ☐ 5-9 years (3)
- ☐ 10 years or more (4)

4. What are your main work assignments? *Please describe in your own words.* (occ_tasks)
(text)

____

____

____

5. Please state the name, department and address of your work place: *If your work place consists of more than one building, please state the name of the building in which you spend most of your time.* (occ_address)

(text)

____

____

____

6. How far is it between your home and your work place? *Please estimate the actual travel distance, not the air distance. Give as accurate an estimate as possible.* (occ_distance)

- ☐ Less than 500 metres (1)
- ☐ 500 metres or more, but less than 1 kilometre (2)
- ☐ 1 kilometre or more, but less than 2 kilometres (3)
- ☐ 2 kilometres or more, but less than 5 kilometres (4)
- ☐ 5 kilometres or more, but less than 10 kilometres (5)
- ☐ 10 kilometres or more, but less than 20 kilometres (6)
- ☐ 20 kilometres or more, but less than 50 kilometres (7)
- ☐ 50 kilometres or more, but less than 100 kilometres (8)
- ☐ 100 kilometres or more (9)

7. How long have you been working at the work place stated in question number 5? (occ_address_yrs)

- ☐ Less than 1 year (1)
- ☐ 1-4 years (2)
- ☐ 5-9 years(3)
- ☐ 10 years or more (4)

Here are some questions regarding last week, (or, if your last week was different from an ordinary one, regarding the last ”ordinary” week):

8. How much did you work last week, or during the last ”ordinary” week? (occ_time_perwk)

- ☐ Full time (1)
- ☐ 75% or more, but less than full time (2)
- ☐ Half time or more, but less than 75% (3)
- ☐ 25% or more, but less than half time (4)
- ☐ Less than 25% (5)

9. What were your working hours? (occ_timeofday)

- ☐ Daytime (1)
- ☐ Daytime + on call-work (2)
- ☐ Mostly evenings/nights (3)
- ☐ Shift work, two-shift (4)
- ☐ Shift work, three-five shift (5)
- ☐ Other (6)

10. Which one was your main means of travel when travelling to work last week (or in the last ”ordinary” week)? *Only give one alternative.* (travel_mode)

- ☐ By foot ⇒ *Go to question **12*** (1)
- ☐ By bike ⇒ *Go to question **12*** (2)
- ☐ Travelled alone or with a family member by car ⇒ *Go to question **12*** (3)
- ☐ Travelled by car together with others (e g car pool or by taxi) ⇒ *Go to question **12*** (4)
- ☐ Travelled by bus or train ⇒ *Go to question 11* (5)
- ☐ Travelled in other ways ⇒ *Go to question **12*** (6)

11. How crowded was it usually on the bus/train to your place of work last week? *If it varied, please state how it usually was as regards the distance and direction where most people travelled.* (travel_crowdedness)

☐ All seats were taken– and people had to stand up(1)

☐ All seats were taken but nobody had to stand up (2)

☐ There were a few free seats (3)

☐ There were plenty of free seats (4)

12. On an ordinary day last week (or the last ”ordinary” week), how much time did you spend <u>outdoors</u> when at work? (occ_outside_hrsperd)

☐ 0 hours (1)

☐ More than 0 hours, but less than 1 hour (2)

☐ 1 hour or more, but less than 3 hours(3)

☐ 3 hours or more, but less than 5 hours(4)

☐ 5 hours or more, but less than 7 hours(5)

☐ 7 hours or more (6)

Being outdoors in a closed, heated cabin or equivalent, i e as a driver, machine operator or conductor, counts as being indoors. Construction workers in a ”sealed” house are counted as being indoors, other construction work is counted as being outdoors.

13. When working indoors, do you have a permanent workspace where you spend at least 25% of your working time? *A driver’s cabin and equivalent also counts as a workspace.* (place_same)

☐ Yes ⇒ *Go to question* ***14*** (1)

☐ No, I usually move between several different premises, mainly within the same place of work.
⇒ *<u>Jump to question</u>* ***20*** (2)

☐ No, most of the time I move between several different premises, both within and outside of the place of work
⇒ *<u>Jump to question</u>* ***50*** (3)

☐ No, I work almost only outdoors ⇒ *<u>Jump to question</u>* ***47*** (4)

14. What is the workspace like where you spend at least 25% of your working time?
"Workspace" here refers to <u>*the entire*</u> *room in which you work, even if you have only a smaller part of it at your disposal. Tick the alternative closest to the truth.* (place_type)

☐ A driver's cabin/booth or equivalent ⇒ *<u>Jump to question</u>* ***33*** (1)

☐ <u>Own</u> room/premises where you work alone. *This does not rule out occasional visitors to the premises* ⇒ *<u>Jump to question</u>* ***16*** (2)

☐ Room/premises shared with <u>one</u> other person ⇒ *Go to question* ***15*** (3)

☐ Room/premises shared with 2 other persons or more, but fewer than 5 persons ⇒ *Go to question* ***15*** (4)

☐ Room/premises shared with 5 other persons or more, but fewer than 10 persons ⇒ *Go to question* ***15*** (5)

☐ Room/premises shared with 10 other persons or more, but fewer than 20 persons ⇒ *Go to question* ***15*** (6)

☐ Room/premises shared with 20 other persons or more. ⇒ *Go to question* ***15*** (7)

15. Is there any partition between you and the person/persons working closest to you?
(p1_divider)

☐ Yes (1)

☐ No (0)

With "partition" we refer to a divider, screen, a piece of furniture, machine or similar which stops you from seeing your "neighbour" when sitting on a chair.

16. Approximately how large is the workspace you referred to in question 14? *Give as accurate an estimate as possible.* (p1_size)

- ☐ Less than 10 square metres (1)
- ☐ 10 square metres or more, but less than 20 square metres (2)
- ☐ 20 square metres or more, but less than 50 square metres (3)
- ☐ 50 square metres or more, but less than 100 square metres (4)
- ☐ 100 square metres or more, but less than 500 square metres (5)
- ☐ 500 square metres or more, but less than 1000 k square metres (6)
- ☐ 1000 square metres or more (7)

17. Including yourself, approximately how many persons <u>dwelled more than just temporarily</u> (for example worked, studied, or were taken care of) in your workspace on an ordinary day last week (or the last "ordinary" week)? *Give as accurate an estimate as possible.* (p1_visitors_int_long)

- ☐ 1 (1)
- ☐ 2 (2)
- ☐ 3-4 (3)
- ☐ 5-9 (4)
- ☐ 10-19 (5)
- ☐ 20-29 (6)
- ☐ 30-49 (7)
- ☐ 50-74 (8)
- ☐ 75-99 (9)
- ☐ 100-149 (10)
- ☐ 150 or more (11)

18. Approximately how many <u>in-house visitors temporarily visited</u> your workspace on an ordinary day last week (or the last "ordinary" week)? Give as accurate an estimate as possible. (p1_visitors_int_short)

- ☐ 0 (1)

☐ 1-4 (2)

☐ 5-9 (3)

☐ 10-19 (4)

☐ 20-49 (5)

☐ 50-99 (6)

☐ 100 or more (7)

With in-house visitors we refer to people working at your place of work, but who do not share work space with you. If an individual makes several visits per day he/she should only be counted once.

19. Approximately how many external visitors temporarily visited your workspace on an ordinary day last week (or the last "ordinary" week) *Of course it is difficult to give the exact number, but please give as accurate an estimate as possible.* (p1_visitors_ext_short)

☐ 0 ⇒ *Jump to question **25*** (1)

☐ 1-4 ⇒ *Jump to question **25*** (2)

☐ 5-9 ⇒ *Jump to question **25*** (3)

☐ 10-19 ⇒ *Jump to question **25*** (4)

☐ 20-49 ⇒ *Jump to question **25*** (5)

☐ 50-99 ⇒ *Jump to question **25*** (6)

☐ 100-199 ⇒ *Jump to question **25*** (7)

☐ 200-499 ⇒ *Jump to question **25*** (8)

☐ 500-999 ⇒ *Jump to question **25*** (9)

☐ 1000 or more ⇒ *Jump to question **25*** (10)

With external visitors we refer to people who do not work at your place of work but who represent the public (i e guests, customers, clients, patients) or other companies/institutions/authorities than the one you work at. If an individual makes several visits per day he/she should only be counted once.

20. What are the premises, where you usually spend your working hours, like? *Tick the alternative closest to the truth.* (p2_spread)

- ☐ A limited group of rooms connected to each other, e g a care unit, a small office space ⇒ *Go to question* ***21*** (1)
- ☐ Premises that are spread out (e g conference and meeting rooms in different departments, premises for in-house property maintenance – i e premises between which you have to transport yourself for at least one minute) ⇒ *Jump to question* ***25*** (2)
- ☐ Extensive premises that are both connected and spread out (e g cleaning work) ⇒ *Jump to question* ***25*** (3)

21. Approximately how large in total is the workspace you dwelled in on an ordinary day last week (or the last "ordinary" week)? *Give as accurate an estimate as possible* (p2_size_m2)

- ☐ Less than 50 square metres (1)
- ☐ 50 square metres or more, but less than 100 square metres (2)
- ☐ 100 square metres or more, but less than 500 square metres(3)
- ☐ 500 square metres or more, but less than 1000 square metres (4)
- ☐ 1000 square metres or more (5)

22. Including yourself, approximately how many persons dwelled more than just temporarily within the total area that you referred to in question 21? *This still concerns an ordinary day during last week (or the last "ordinary" week). Give as accurate an estimate as possible.* (P2_persons_long)

- ☐ 1 (1)
- ☐ 2 (2)
- ☐ 3-4 (3)
- ☐ 5-9 (4)
- ☐ 10-19 (5)
- ☐ 20-29 (6)
- ☐ 30-49 (7)
- ☐ 50-74 (8)
- ☐ 75-99 (9)
- ☐ 100-149 (10)
- ☐ 150 or more (11)

23. Approximately how many <u>in-house visitors temporarily visited</u> the total workspace on an ordinary day last week (or the last ”ordinary” week)? *Give as accurate an estimate as possible.* (P2_persons_int_short)

- ☐ 0 (1)
- ☐ 1-4 (2)
- ☐ 5-9 (3)
- ☐ 10-19 (4)
- ☐ 20-49 (5)
- ☐ 50-99 (6)
- ☐ 100 or more (7)

With <u>in-house visitors</u> we refer to people people wotking at your company/institution/authority but who do not have their work space within the area referred to in question 21. If an individual makes several visits per day he/she should only be counted once.

24. Approximately how many <u>external visitors</u> temporarily visited the total workspace on an ordinary day last week (or the last ”ordinary” week)? *Of course it is difficult finding out the exact number, but please give as accurate an estimate as possible.* (P2_persons_ext_short)

- ☐ 0 (1)
- ☐ 1-4 (2)
- ☐ 5-9 (3)
- ☐ 10-19 (4)
- ☐ 20-49 (5)
- ☐ 50-99 (6)
- ☐ 100-199 (7)
- ☐ 200-499 (8)
- ☐ 500-999 (9)
- ☐ 1000 or more (10)

With <u>external visitors</u> we refer to people who do not work at your place of work but who represent the public (i e guests, customers, clients, patients) or other companies/institutions/authorities than the one you work at. If an individual makes several visits per day he/she should only be counted once.

25. According to your own assessment, from which decade does the building in which you normally spent your working hours on an ordinary day last week originate (or the last "ordinary week)? *If you do not know, please give as accurate an estimate as possible.* (p2_building_decade)

- ☐ From the 2000s or the 2010s (1)

- ☐ From the 1990s (2)
- ☐ From the 1980s (3)
- ☐ From the 1970s (4)
- ☐ From the 1960s(5)
- ☐ From the 1950s (6)
- ☐ From the 1940s (7)
- ☐ Cannot answer (998)

> *With "originate" we refer to the construction year. If the building has been completely renovated since then, then state the decade when the last complete restoration took place.*

26. Regarding the workspace you have referred to in your previous answers, approximately how high is the ceiling height?
Give as accurate an estimate as possible. (p2_ceiling_height)

- ☐ Approximately lower than 3 metres (1)
- ☐ Approximately 3-5 metres (2)
- ☐ Approximately higher than 5 metres (3)

27. What is the floor like? (p2_floor_hardness)

- ☐ Hard material (wood/parquet, stone, concrete, laminate, linoleum, cork, vinyl, plastic, rubber or equivalent) with no carpets (1)
- ☐ Hard material with loose textile carpets (2)
- ☐ Fitted carpet (3)
- ☐ Hard material in some places and fitted carpet in others(4)
- ☐ Other (5)

28. How is the cleaning managed? *Tick the boxes matching the frequency of the floor cleaning, the wiping of work surfaces, door handles, etc. Tick one box in each row.*

	Every day(1)	Not every day but more than once a week(2)	Once a week (3)	More rarely than once a week(4)	Don't know(998)
Floor cleaning(p2_cleaning_floor)	☐	☐	☐	☐	☐
Wiping of work surfaces, door handles etc(p2_cleaning_workspace)	☐	☐	☐	☐	☐

29. Do you share your work surface (a desk or equivalent) with others at your work place? (p2_share_table)

☐ Yes, I have a work surface at work and share it with one co-worker (1)

☐ Yes, I have a work surface at work and share it with several co-workers(2)

☐ No, I do have a work surface at work but I don't share with anyone (3)

☐ No, I don't have a work surface at work (4)

30. Do you share a telephone with others at your work place? (p2_share_phone)

☐ Yes, I share my telephone with one co-worker (1)

☐ Yes, I share my telephone with several co-workers (2)

☐ No, I do have a telephone at work but I do not share it with anyone (3)

☐ No, I don't have a telephone at work (4)

31. Do you share your keyboard (computer, cash register or equivalent) with others at your work place? (p2_share_keyboard)

☐ Yes, I share a keyboard with one co-worker (1)

☐ Yes, I share a keyboard with <u>several</u> co-workers (2)

☐ No, I do use a keyboard at work but I do not share with anyone (3)

☐ No, I do not use a keyboard at work (4)

32. Do you share a console or equivalent with others at your work place? (p2_share_console)

☐ Yes, I share a console with <u>one</u> co-worker ⇒ *<u>Jump to question</u>* ***37*** (1)

☐ Yes, I share a console with <u>several</u> co-workers ⇒ *<u>Jump to question</u>* ***37*** (2)

☐ No, I do use a console at work but I do not share it with anyone ⇒ *<u>Jump to question</u>* ***37*** (3)

☐ No, I do not use a console at work ⇒ *<u>Jump to question</u>* ***37*** (4)

33. Approximately how large is your workspace? *For example, if you are a bus or taxi driver then estimate the whole passenger compartment. Give as accurate an estimate as possible.* (p3_size)

☐ Less than 2 square metres (1)

☐ 2 square metres or more, but less than 5 square metres (2)

☐ 5 square metres or more, but less than 10 square metres (3)

☐ 10 square metres or more, but less than 20 square metres (4)

☐ 20 square metres or more, but less than 50 square metres (5)

☐ 50 square metres or more (6)

34. Do you share a driving seat, console or equivalent with others? (p3_share_console)

☐ Yes, I share a driving seat/console with <u>one</u> person (1)

☐ Yes, I share a driving seat/ console with several persons (2)

☐ No, I do have a driving seat/ console, but do not share it with anyone (3)

☐ No, I do not use a driving seat/ console at work (4)

35. Approximately how many <u>in-house visitors</u> <u>temporarily</u> visited your workspace during an ordinary day last week (or the last "ordinary" week)? *Give as accurate an estimate as possible.* (p3_visitors_int_short)

☐ 0 (1)

☐ 1-4 (2)

☐ 5-9 (3)

☐ 10-19 (4)

☐ 20-49 (5)

☐ 50-99 (6)

☐ 100 or more (7)

With <u>in-house visitors</u> we refer to people working at your place of work, but who do not share work space with you. If an individual makes several visits per day he/she should only be counted once.

36. Approximately how many <u>external visitors</u> temporarily visited your workspace during an ordinary day last week (or the last "ordinary" week)? *Give as accurate an estimate as possible.* (p3_visitors_ext_short)

☐ 0 (1)

☐ 1-4 (2)

☐ 5-9 (3)

☐ 10-19 (4)

☐ 20-49 (5)

☐ 50-99 (6)

☐ 100-199 (7)

☐ 200-499 (8)

☐ 500 or more (9)

With <u>external visitors</u> we refer to people who do not work at your place of work, but who represent the public (i e passengers, customers, patients). If an individual makes several visits per day he/she should only be counted once

37. What kind of ventilation do you have at your work place? (p3_ventilation)

- ☐ Natural draft, e g no ventilator affects the ventilation (1)
- ☐ Mechanical, e g controlled by ventilator (2)
- ☐ No ventilation, e g only the possibility of opening a window (3)
- ☐ Do not know (998)

38. In summer, is there any refrigerated supply air in your workspace? (p3_ventilation_cool)

- ☐ Yes (1)
- ☐ No (0)
- ☐ Do not know (998)

39. What is your experience of the quality of the ventilation? (p3_ventilation_quality)

- ☐ Good (1)
- ☐ Could be better (2)
- ☐ Poor (3)
- ☐ Cannot answer (998)

40. How do you experience the air temperature in your workspace? *Only tick one alternative on each row.*

	Mostly too warm (1)	Mostly adequate(2)	Mostly too cold (3)	It varies a lot – sometimes too warm, sometimes too cold(4)	Cannot answer(998)
During summer months (p3_air_temperature_summer)	☐	☐	☐	☐	☐
During winter months (p3_air_temperature_winter)	☐	☐	☐	☐	☐

41. How often do you perceive the air in your workspace as being dry? *Only tick one alternative in each row*

	Never (1)	In exceptional cases (2)	Quite often (3)	Always(4)	Cannot answer (998)
During summer months (p3_air_humidity_summer)	☐	☐	☐	☐	☐
During winter months (p3_air_humidity_winter)	☐	☐	☐	☐	☐

42. How often do you perceive the air in your workspace as stuffy? (p3_air_stuffy)

- ☐ Never (1)
- ☐ In exceptional cases (2)
- ☐ Quite often (3)
- ☐ Always (4)

43. Do you experience any problems with damp at your work place? (p3_humidity_problems)

- ☐ No, certainly not (1)

☐ Possibly (2)

☐ Yes, probably (3)

☐ Yes, definitively (4)

44. How often can you smell mould (like in an underground storehouse) at your work place? (p3_mold_smell)

☐ Never (1)

☐ In exceptional cases (2)

☐ Quite often (3)

☐ Always (4)

45. Is there any growth of mould and/or damp spots in the ceiling, on the walls or on the floor of your workspace? (p3_mold_visible)

☐ Have never noticed anything like that (1)

☐ It has occurred only the once (2)

☐ It has come and gone (3)

☐ It is there all the time (4)

46. During winter months, how often is there a substantial amount of vapour/condensation on the inside of the windows in your workspace? (p3_winter_condensation)

☐ Never ⇒ <u>*Jump to question*</u> ***49***(1)

☐ In exceptional cases ⇒ <u>*Jump to question*</u> ***49*** (2)

☐ Quite often ⇒ <u>*Jump to question*</u> ***49***(3)

☐ Always ⇒ <u>*Jump to question*</u> ***49***(4)

☐ Do not know ⇒ <u>*Jump to question*</u> ***49***(5)

With "substantial amount of vapor/condensation" we refer to the vapor or water drops reaching at least 5 cm in on the window pane.

47. When working in freezing-temperature, with no or only a light wind, how often do you freeze to the degree of starting to feeling numb in the nose and/or fingers? (p4_nose_numb_zero)

- ☐ Never (1)
- ☐ In exceptional cases (2)
- ☐ Less than 10% of the working hours outdoors (3)
- ☐ 10% or more of the working hours outdoors, but less than 50% (4)
- ☐ 50% or more of the working hour outdoors, but not always (5)
- ☐ Always (6)

48. When working in −10° C, with no or only a light wind, how often do you freeze so much that you feel numb in the nose and/or fingers? (p4_nose_numb_10)

- ☐ Never (1)
- ☐ In exceptional cases (2)
- ☐ Less than 10% of the working hours outdoors(3)
- ☐ 10% of the working hours outdoors, but less than 50% (4)
- ☐ 50% of the working hours outdoors, but not always (5)
- ☐ Always (6)

49. How far is it from the place where you usually work to the closest wash room/sink? (p4_sink_distance)

- ☐ Less than 20 metres (1)
- ☐ 20-49 metres (2)
- ☐ 50-99 metres (3)
- ☐ 100 metres or more (4)
- ☐ It varies since I move around in large areas (5)

With <u>wash room</u> /sink we refer to a bath room or wash room twhere you can wash your hands with soap and hot running water.

50. Approximately how many persons share the wash room/sink that you normally use?
If using several different wash rooms/sinks, please consider the one you most <u>usually use</u>.
(p4_sink_perperson)

- ☐ 1-4 persons (1)

☐ 5-9 persons (2)

☐ 10-19 persons (3)

☐ 20-49 persons (4)

☐ 50 or more persons (5)

With wash room /sink we refer to a bath room or wash room twhere you can wash your hands with soap and hot running water.

51. Do the public also use this wash room/sink? (p4_sink_public)

☐ Generally speaking, never (1)

☐ It happens once or a few times per week (2)

☐ It happens daily or almost daily (3)

☐ It happens up to 10 times a day (4)

☐ It happens more than 10 times a day (5)

With the public we refer to for example customers temprarily visiting your place of work.

52. Do you have access to soap or equivalent in the wash room/sink? (p4_sink_soap)

☐ Always (1)

☐ Most of the time (2)

☐ It has often run out (3)

☐ There is never or almost never any soap in the wash room/sink ⇒ *Jump to question* ***54***(4)

53. What type of soap is it usually? (p4_sink_soap_type)

☐ Liquid soap in a dispenser (1)

☐ Other type of soap (2)

54. How can one dry the hands in the wash room/sink? *Tick all alternatives that are available in the wash room/sink.*

☐ Paper towels or other disposable towels (p4_sink_dry_type_1)

☐ Towel made of fabric, in a dispenser presenting a clean section for each new user (p4_sink_dry_type_2)

☐ Ordinary towel made of fabric for common multiple use (like at home) (p4_sink_dry_type_3)

☐ Electric hand drier (p4_sink_dry_type_4)

☐ Other type of towel/hand drying device (p4_sink_dry_type_5)

55. Wash rooms may be managed in varying ways. Is the opportunity of drying your hands in the wash room/sink that you normally use satisfactory? (p4_sink_dry_available)

☐ Yes, always. (1)

☐ Yes, most of the time (2)

☐ No, the hand drying-opportunities are often limited due to missing or dirty towels and/or the hand drier being out of order (3)

☐ No, there is almost never anything to dry your hands with (4)

56. Do you have access to hand disinfectant in the wash room/sink? (p4_sink_disinfectant)

☐ Yes, always (1)

☐ Yes, most of the time (2)

☐ Yes, occasionally (3)

☐ No, never or almost never (4)

57. Where did you usually have your lunch during the weekdays of the last week (or the last "ordinary" week)? (p4_food_place)

☐ In the canteen/restaurant at your place of work (1)

- ☐ In a cafe/restaurant outside your place of work (2)
- ☐ In the coffee room or in a smaller staff room at your work place(3)
- ☐ At your own work space (4)
- ☐ At home (5)
- ☐ In other place (6)
- ☐ Did not have any lunch last week(7)

58. How are the hot dishes normally being served in the canteen at your work place? *If there is more than one canteen, consider the canteen that you visit most of the time.* (p4_food_method)

- ☐ It is handed out by the waiting staff (1)
- ☐ You serve yourself from a buffet (2)
- ☐ It is being served in other ways (3)
- ☐ There is no canteen at my place of work (4)
- ☐ I do not know what it is like at the canteen because I almost never go there (5)

59. Do you normally have "coffee breaks" in the staff room/coffee room? (p4_food_break)

- ☐ Yes, several times a day (1)
- ☐ Yes, once a day (2)
- ☐ Yes, but not every day (3)
- ☐ Rarely or never ⇒ *Jump to question* ***64*** (4)

60. What standard of equipment does the staff room/coffee room have? *Tick all the alternatives matching the staff room/coffee room in which you most often have your coffee breaks.*

- ☐ Kitchen sink with hot and cold running water (p4_food_equipment_1)

- ☐ Fridge (p4_food_equipment_2)
- ☐ Dish washer (p4_food_equipment_3)
- ☐ Hotplates/hob (p4_food_equipment_4)
- ☐ ”Manual” coffee maker (p4_food_equipment_5)
- ☐ Coffee machine (p4_food_equipment_6)
- ☐ Kettle (p4_food_equipment_7)
- ☐ Microwave oven (p4_food_equipment_8)

61. How is the washing up taken care of in the staff room/coffee room?(p4_food_dishes)

- ☐ There is almost always access to clean dishware or disposable cups (1)
- ☐ There is sometimes access clean dishware or disposable cups (2)
- ☐ There is almost never access to clean dishware or disposable cups (3)

62. How do you experience the level of hygiene in the staff room/coffee room? (p4_food_cleaning)

- ☐ Very good (1)
- ☐ Good (2)
- ☐ Neither good nor poor (3)
- ☐ Poor (4)
- ☐ Very poor (5)

63. Is there a food buffet in the staff room/coffee room? (p4_food_buffet)

- ☐ Yes, every day or almost every day (1)
- ☐ Yes, more than once a week, but not every day (2)
- ☐ Yes, once a week(3)
- ☐ Yes, but mostly occasionally(4)
- ☐ Rarely or never (5)

With food buffet we refer to food, sandwich spread or other finger food being on a table and that everybody serves themselves.

64. Has any of the following air contaminations occurred at your work place during the last six months? *State how many hours you have been exposed in an ordinary week. Tick one alternative in each row. If you alternate between different work places, please state how it was altogether in all work places you have been at during an ordinary week.*

	Never or almost not at all(1)	About 4 hours per week(2)	About 10 hours per week(3)	20 hours or more per week(4)
Motor fumes (from motor vehicles or working machines – this does not refer to journeys to and from work) (p4_contamination_engine)	☐	☐	☐	☐
Dust (e. g. grinding dust, whirling dust, construction dust or wood dust) (p4_contamination_dust)	☐	☐	☐	☐
Smoke (e. g. welding fumes, combustion smoke or solder smoke – this does not refer to tobacco smoke) (p4_contamination_smoke)	☐	☐	☐	☐
Solvent (e. g. paint, glue or detergents) (p4_contamination_solvent)	☐	☐	☐	☐
Other chemical products (e. g. hair dresser's chemicals, car-care products or dental care products) (p4_contamination_chemicals)	☐	☐	☐	☐
Tobacco smoke from others (i e passive smoking) (p4_contamination_tobacco)	☐	☐	☐	☐

The questionnaire is now finished. Please return it in the post-free self addressed envelope enclosed. Thank you for your answers!
