# Supplementary material for: Cohort profile: Studies of Work Environment and Disease Epidemiology-Infections (SWEDE-I), a prospective cohort on employed adults in Sweden
Source: PLoS One. 2019 May 15;14(5):e0217012. doi: 10.1371/journal.pone.0217012 (PMC6519895; doi:10.1371/journal.pone.0217012)
Supplement: S2 File — (PDF) [file pone.0217012.s002.pdf]

2. Specific conditions at work

1. **Do you use protective clothing at work?** *Tick all alternatives that match how it was on an ordinary day last week (or the last "ordinary" week)*.

   - ☐ No, I only use my own private clothes at work (protective_clothing_1)
   - ☐ Yes, I use specific work clothes, uniform or profile clothes at work and change to private clothes before I go home (protective_clothing_2)
   - ☐ Yes, I use specific work clothes, uniform or profile clothes at work and go home in these clothes (protective_clothing_3)
   - ☐ Yes I use protective clothing or an overall over my private clothes or work clothes (protective_clothing_4)
   - ☐ Yes, I use other types of protective clothing (protective_clothing_5)

2. **Do you regularly use other protective equipment in your work?** *Tick all the alternatives that match how it was during an ordinary day last week (or the last "ordinary" week)*.

   - ☐ Yes, protective gloves/working gloves (protective_other_1)
   - ☐ Yes, cap/helmet/hair protection (protective_other_2)
   - ☐ Yes, protective goggles (protective_other-3)
   - ☐ Yes, protective mask/respiratory protective equipment (protective_other_4)
   - ☐ Yes, safety shoes/work shoes/-boots (protective_other_5)
   - ☐ Yes, other protective equipment (protective_other_6)
   - ☐ No, I do not usually use any protective equipment (protective_other_7)

3. **Does your job imply that you get as dirty as having to shower or have a bath at work or immediately after arriving home?** (dirty)

   - ☐ Yes, every day (1)
   - ☐ Yes, at least once a week (2)
   - ☐ Yes, but more rarely (3)
   - ☐ No, never or almost never(4)

4. Does your job involve serving the public face to face, more than just temporarily? *With ”public” we refer to customers, clients, patients, passengers, advice seekers. With ”face to face” we refer to personal encounters at a distance less than 1½ metres.*(public_contact)

- ☐ No ⇒ *Jump to question* ***11 (0)***
- ☐ Yes, but less than 25% of my working time ⇒ *Go to question* ***5 (1)***
- ☐ Yes, 25% or more of my working time, but less than 50% ⇒ *Go to question* ***5 (2)***
- ☐ Yes, 50% or more of my working time, but less than 75% ⇒ *Go to question* ***5 (3)***
- ☐ Yes, 75% or more of my working time ⇒ *Go to question* ***5 (4)***

5. On an ordinary day last week (or the last ”ordinary” week), approximately how many encounters like that did you have? *Give as accurate an estimate as possible.*(public_num)

- ☐ Less than 10(1)
- ☐ 10-29(2)
- ☐ 30-49(3)
- ☐ 50-69(4)
- ☐ 70-89(5)
- ☐ 90-109(6)
- ☐ 110-199(7)
- ☐ 200 or more(8)

We refer to encounters such as described in the previous question.

6. How did these encounters take place?

- ☐ Mostly over a counter, conveyor belt, desk or other work surface (public_type_1)
- ☐ Mostly at speaking distance without intermediary work surface (public_type-2)
- ☐ In other ways (public_type_3)

7. Was there usually any partition (glass pane, shutter, or similar) between you? (public_divider)

- ☐ Yes(1)
- ☐ No(0)

8. **Did you usually exchange money, payment cards or similar?** (public_money)

☐ Yes, and everything was handed over from hand to hand (1)

☐ Yes, bank notes were handed over from hand to hand, but coins and payment cards were handled only by the customer/client and not by me (2)

☐ Yes, bank notes as well as coins and payment cards were handled only by the customer/client and not by me (3)

☐ No, no money or payment cards were exchanged(4)

9. **How often were other items (i e products, parcels, documents, tickets) handed over to you from hand to hand?** (public_parcels)

☐ Almost never (1)

☐ At up to 1/3 of all encounters (2)

☐ At 1/3 or more of all encounters, but less than 2/3(3)

☐ At 2/3 or more of all encounters (4)

☐ At generally every encounter(5)

10. **How often were there handshakes at the encounters?** (public_handshake)

☐ Almost never (1)

☐ At up to 1/3 of all encounters (2)

☐ At 1/3 or more of all encounters, but less than 2/3 (3)

☐ At 2/3 or more of all encounters(4)

☐ At generally every encounter (5)

With handshake we refer to shaking hands.

11. **Do you work with patients within health care or similar areas, for example ambulance service?** (public_caregiver)

☐ Yes ⇒ *Go to question* ***12(1)***

☐ No ⇒ *Jump to question* ***13(0)***

12. How many of the patients that you were in close contact with during an ordinary working period last week (or the last “ordinary” week) showed signs of cold or other respiratory ill-health? *Give as accurate an estimate as possible.*(public_caregiver_flu)

- ☐ 0 (1)
- ☐ 1(2)
- ☐ 2-4(3)
- ☐ 5-9(4)
- ☐ 10-19(5)
- ☐ 20 or more (6)

> *With ”close contact” we refer to having a distance between you of* ***<u>less than a meter</u>*** *for* ***<u>more than 1 minute</u>***.

13. Does your work involve having regular contact with children below 13 years of age? (public_children)

- ☐ Yes, most of my working time ⇒ *Go to question* ***14(1)***
- ☐ Yes, but only sporadically ⇒ *Go to question* ***14(2)***
- ☐ No ⇒ *<u>Jump to question</u>* ***15(0)***

> *With ”close contact” we refer to having a distance between you of* ***<u>less than a meter</u>*** *for* ***<u>more than 1 minute</u>***.

14. During an ordinary working period last week (or the last ”ordinary” week), how many children of different ages were you in close contact with?
Every single child should only be counted once.
Of course it is hard to know the exact number, and we therefore ask you to reflect for a while. Try and give as accurate an estimate as possible.
Only tick one in each row.

	0(1)	1(2)	2-4(3)	5-9(4)	10-19(5)	20-29(6)	30-44(7)	45+(8)
**Children of age <u>0-2 years</u>** (public_children_0to2yrs)	☐	☐	☐	☐	☐	☐	☐	☐
**Children of age <u>3-6 years</u>** (public_children_3to6yrs)	☐	☐	☐	☐	☐	☐	☐	☐
**Children of age <u>7-13 years</u>** (public_children_7to13yrs)	☐	☐	☐	☐	☐	☐	☐	☐

15. Approximately how many times during an ordinary day last week (or the last ”ordinary” week) did you pass closed doors, and had to touch the handle or other opening mechanism to open them? *Give as accurate an estimate as possible.*
(objects_door)

- ☐ 0-9 door passageways(1)
- ☐ 10-49 door passageways (2)
- ☐ 50-99 door passageways (3)
- ☐ 100 door passageways (4)

Only count the doors you have passed whilst at work. Do not count automatic doors that open without any body contact,, car doors or other doors on vehicles, and not inspection panels and other entrances to technical areas.

16. How often do you use a closed elevator at work? (objects_elevetor)

- ☐ Never or almost never ⇒ *Jump to question* ***18(1)***
- ☐ Every now and then, but not daily ⇒ Go to question ***17(2)***
- ☐ 1-4 times a day ⇒ Go to question ***17(3)***
- ☐ 5-9 times a day ⇒ Go to question ***17(4)***
- ☐ 10 times a day or more ⇒ Go to question ***17(5)***

17. When using an elevator at work, how many persons are there usually in the elevator? (objects_elevetor_persons)

- ☐ I am almost always by myself(1)
- ☐ There are sporadically some fellow passengers, a few persons(2)
- ☐ Often or almost always some fellow passengers, a few persons (3)
- ☐ Often or almost always some fellow passengers, many persons(4)

18. During an ordinary work period last week (or the last "ordinary" week), how many times did you wash your hands? *Of course it is difficult to know the exact number, but please give an estimate.* (hand_wash)

- ☐ 0(1)
- ☐ 1(2)
- ☐ 2-4(3)
- ☐ 5-9(4)
- ☐ 10-19(5)
- ☐ 20 or more (6)

19. When you washed your hands at work, how long did it take from when you started to lather and wet them until you stopped washing them? *Give as accurate an estimate as possible.*(hand_wash_time)

- ☐ Usually less than 5 seconds (1)
- ☐ 5-9 seconds(2)
- ☐ 10-19 seconds(3)
- ☐ 20-29 seconds(4)
- ☐ 30-39 seconds (5)
- ☐ 40 seconds or more(6)

20. How did you dry your hands? (hand_wash_dry)

- ☐ With paper towels or other disposable towels(1)
- ☐ Towel made of fabric, in a dispenser presenting a clean section for each new user (2)
- ☐ Ordinary towel made of fabric for common multiple use (like at home)(3)
- ☐ Electric hand drier(4)
- ☐ Other type of towel/hand drying device (5)
- ☐ I dried my hands on the trouser leg or equivalent (6)
- ☐ I let my hands self-dry (7)
- ☐ I did not wash my hands at work that week.

21. During <u>an ordinary working period</u> last week (or the last "ordinary" week), how many times did you use disinfectant on your hands? *It is of course hard to know exactly, but please give an estimate.* (hand_disinfectant)

- ☐ 0(1)
- ☐ 1(2)
- ☐ 2-4(3)
- ☐ 5-9(4)
- ☐ 10-19(5)
- ☐ 20 or more (6)

22. How physically strenuous is your work usually? (phys_level)

- ☐ Light, mostly sitting still ⇒ *<u>Jump to question</u>* ***26(1)***
- ☐ Light, but I move around a bit ⇒ *Go to question* ***23(2)***
- ☐ Quite strenuous ⇒ *Go to question* ***23(3)***
- ☐ Very strenuous ⇒ *Go to question* ***23(4)***

23. In what way/ways do you move about/make physical effort at your work? *Tick one or more alternatives.*

- ☐ Transportation, i e walking, running, riding a bike, climbing or swimming(phys_type_1)
- ☐ Muscle power, i e lifting, wrenching, pushing, squeezing or wringing(phys_type_2)
- ☐ Other type of physical effort (phys_type_3)

24. In total during the last week (or the last ”ordinary” week), for how long were you __short of breath__ due to physical effort at work? *Give as accurate an estimate as possible.* (phys_shortofbreath)

- ☐ I was never short of breath due to work(1)
- ☐ Less than 5 minutes(2)
- ☐ Five minutes up to half an hour(3)
- ☐ Half an hour up to one hour(4)
- ☐ One hour up to 6 hours (5)
- ☐ 6 hours or more(6)

25. In total during the last week (or the last ”ordinary” week), for how long were you __sweaty__ due to physical effort at work? *Give as accurate an estimate as possible.* (phys_sweat)

- ☐ I was never sweaty due to physical effort at work(1)
- ☐ Less than 5 minutes(2)
- ☐ Five minutes up to half an hour(3)
- ☐ Half an hour up to one hour(4)
- ☐ One hour up to 6 hours(5)
- ☐ 6 hours or more (6)

26. Do you think you have the knowledge and skill to handle your job? *Do you feel overqualified, adequately qualified, or do you feel that you are missing the knowledge needed? Tick the alternatives that best match your opinion.* (cond_qualified)

- ☐ Very overqualified(1)
- ☐ In some respects overqualified (2)
- ☐ Adequately qualified(3)
- ☐ Missing some knowledge needed (4)
- ☐ Missing a lot of knowledge needed (5)

27. Does your job require that you.... *(tick <u>one</u> alternative in each row)*

	Never or rarely(1)	Sometimes(2)	Often(3)	Always or almost always(4)
**Work very quickly?** (cond_intensity_fast)	☐	☐	☐	☐
**Work very hard?** (cond_intensity_hard)	☐	☐	☐	☐
**Work too much?** (cond_intensity_toomuch)	☐	☐	☐	☐

28. Do you have enough time for your work assignments? (cond_enoughtime)

- ☐ Never/rarely(1)
- ☐ Sometimes(2)
- ☐ Often(3)
- ☐ Always/almost always(4)

29. Does your job involve.... *(tick one alternative in each row)*

	Almost or rarely(1)	Sometimes(2)	Often(3)	Always or almost always(4)
**learning new things?** (cond_repetetive_non)	☐	☐	☐	☐
**doing the same thing over and over again?** (cond_repetitive)	☐	☐	☐	☐

30. Does your job require.... *(tick one alternative in each row)*

	Never or rarely(1)	Sometimes(2)	Ofen(3)	Always or almost always(4)
**...skills?** (cond_demand_skill)	☐	☐	☐	☐
**ingenuity?**(cond_demand_ingenuity)	☐	☐	☐	☐

31. Are the demands at your work conflicting? (cond_conflicting)

- ☐ Never/rarely(1)
- ☐ Sometimes (2)
- ☐ Often(3)
- ☐ Always/almost always (4)

32. Do you have the freedom to decide on what should be carried out in your job? (choice_what)

- ☐ Never/rarely(1)
- ☐ Sometimes(2)
- ☐ Often(3)
- ☐ Always/almost always(4)

33. Do you have the freedom to decide on how it should be carried out? (choice_how)

- ☐ Never/rarely(1)
- ☐ Sometimes(2)
- ☐ Often(3)
- ☐ Always/almost always(4)

34. Can you make at least one private phone call during working hours? (choice_private_call)

- ☐ Yes(1)
- ☐ No(0)

35. Can you have a private visitor for 10 minutes during working hours? (choice_private_visitor)

- ☐ Yes(1)
- ☐ No(0)

36. Can you make a private errand for half an hour during working hours without asking for permission? (choice_private_errand)

- ☐ Yes(1)
- ☐ No(0)

37. Do you have anybody at your place of work that you can.... *(tick one alternative in each row)*

	Yes(1)	Uncertain(2)	No(0)
**..share interests/experiences with?** (mate_interest)	☐	☐	☐
**..turn to with everyday troubles?** (mate_troubles)	☐	☐	☐
**..be confidential with?** (mate_confidential)	☐	☐	☐

38. If your work assignments are difficult, do you then have the opportunity of receiving advice and help at your work place ? (task_advice)

- ☐ Never(1)
- ☐ Rarely(2)
- ☐ Often(3)
- ☐ Always/almost always(4)

39. Does it ever happen that you are subjected to unfair treatment or collide with a team leader/boss at your place of work? (conflict_boss)

- ☐ Never(1)
- ☐ Rarely (2)
- ☐ Often(3)
- ☐ Always/almost always(4)

40. Does it ever happen that you are subjected to unfair treatment or collide with a colleague at your place of work? (conflict_colleague)

- ☐ never(1)
- ☐ rarely(2)
- ☐ Often(3)
- ☐ Always/almost always(4)

41. Work places may differ regarding the ”presence when ill”, e g people working despite being ill. How is it usually at the work place where you normally work? *If you work in several places, state how you think that it is at the place where you work most of the time.* (sickleave)

- ☐ Sick leave is common(1)
- ☐ Sick leave is uncommon(2)
- ☐ Don’t know/don’t want to answer(3)

The questionnaire is now finished. Please return it in the post-free self addressed envelope enclosed. Thank you for your answers!
