# Supplementary material for: Cohort profile: Studies of Work Environment and Disease Epidemiology-Infections (SWEDE-I), a prospective cohort on employed adults in Sweden
Source: PLoS One. 2019 May 15;14(5):e0217012. doi: 10.1371/journal.pone.0217012 (PMC6519895; doi:10.1371/journal.pone.0217012)
Supplement: S3 File — (PDF) [file pone.0217012.s003.pdf]

3. About you, your family and your contacts

Why do we ask about your family when we are interested in contagion at work? Well, the family is the most common place for spread of infection. It is possible that people having the kind of work conditions we suspect can increase the risk of contagion ALSO have a higher risk for contagion outside of work (for example having kids at nursery). But if we gain knowledge of that risk we can ”sort it out". Then we are able to more accurately estimate the risk of being infected at work.

To begin with are some questions about the household you live in. If you live in different households, we ask you to refer to the one where you live most of the time.

1. How do you live? *Choose the most accurate alternative. (home_type)*

- ☐ Lodger (with parents, relative or landlord)(1)
- ☐ Rented apartment (2)
- ☐ Condominium (3)
- ☐ House/townhouse (4)
- ☐ Other (5)

2. How many rooms except kitchen, hall and bathroom are there in your home? (home_rooms)

- ☐ 1 room (1)
- ☐ 2 rooms (2)
- ☐ 3 rooms (3)
- ☐ 4 rooms (4)
- ☐ 5 rooms (5)
- ☐ 6 rooms (6)
- ☐ 7 rooms (7)
- ☐ 8 rooms or more (8)

3. **How many people live in your household, including yourself?** *If some people just live in your household at certain times (for example children whose separately living parents have shared custody), count the people living in the household more than a fourth of the time (25%). (home_residents)*

- ☐ 1 person ⇒ *If you've ticked this alternative, <u>jump to question</u>* 7 (1)
- ☐ 2 persons (2)
- ☐ 3 persons (3)
- ☐ 4 persons (4)
- ☐ 5 persons (5)
- ☐ 6 persons (6)
- ☐ 7 persons (7)
- ☐ 8 persons or more (8)

4. **How many children <u>below 7</u> years of age live in the household?** *Also count part time residents if they live in the household more than a fourth of the time (25%) If there are no children below 7 years of age in the household, please tick "0 children". (children_0to6yrs)*

- ☐ 0 children ⇒ *If you have ticked this alternative, <u>jump to question</u>* **6** ***(1)***
- ☐ 1 children (2)
- ☐ 2 children (3)
- ☐ 3 children (4)
- ☐ 4 children (5)
- ☐ 5 children (6)
- ☐ 6 children (7)
- ☐ 7 children or more (8)

5. **Where are the child/children during weekdays?**
Tick all alternatives that match the children younger than 7 years living in the household.

- ☐ At pre-school, or school (children_0to6yrs_dwell_1)
- ☐ With a child-minder (children_0to6yrs_dwell_2)
- ☐ At home with a family member or nanny (children_0to6yrs_dwell_3)
- ☐ Other (children_0to6yrs_dwell_4)

6. How many of the following categories live in the household? *Tick one alternative in each row. Also count children living there part time, if they live in the household more than a fourth of the time (25%).*

	0 (1)	1 (2)	2 (3)	3 (4)	4 (5)	5+ (6)
**Children of age <u>7-9 years</u> (children_7to9)**	☐	☐	☐	☐	☐	☐
**Children of age <u>10-12 years</u>(children_10to12)**	☐	☐	☐	☐	☐	☐
**Children of age <u>13-17 years</u> (children_13to17)**	☐	☐	☐	☐	☐	☐

7. What is the total gross income of your household, approximately? *Add up the income for everyone who is providing to the household.(household_income)*

- ☐ Under 100 000 SEK (1)
- ☐ 100 000 – 199 999 SEK (2)
- ☐ 200 000 – 299 999 SEK (3)
- ☐ 300 000 – 399 999 SEK (4)
- ☐ 400 000 – 499 999 SEK (5)
- ☐ 500 000 – 599 999 SEK (6)
- ☐ 600 000 – 799 999 SEK (7)
- ☐ 800 000 – 999 999 SEK (8)
- ☐ 1 000 000 – 1 199 999 SEK (9)
- ☐ 1 200 000 SEK or more (10)

8. What is the <u>highest</u> level of education you have completed? (education)

- ☐ I have not completed any education (1)
- ☐ Elementary school, 6 or 7 years (2)
- ☐ Junior secondary school (3)
- ☐ Elementary school, 9 years (4)
- ☐ Upper secondary school, 2 to 4 years (5)
- ☐ University or college, less than 3 years (6)
- ☐ University or college, 3 years or more (7)
- ☐ Other post-secondary, supplementary education than university or college (8)

9. **What are your body measures?** *Follow the instructions to the right.*
***(body_weight)*=9a,(body_length)=9b,(body_waist)=9c,(body_hip)=9d**

	Weight (kg)				Length (cm)			
	(body_weight_1)	(body_weight_2)	(body_weight_3)	kg	(body_length_1)	(body_length_2)	(body_length_3)	cm
0 (1)		☐	☐			☐	☐	0 (1)
1 (2)	☐	☐	☐		☐	☐	☐	1 (2)
2 (3)	☐	☐	☐		☐	☐	☐	2 (3)
3 (4)		☐	☐			☐	☐	3 (4)
4 (5)		☐	☐			☐	☐	4 (5)
5 (6)		☐	☐			☐	☐	5 (6)
6 (7)		☐	☐			☐	☐	6 (7)
7 (8)		☐	☐			☐	☐	7 (8)
8 (9)		☐	☐			☐	☐	8 (9)
9 (10)		☐	☐			☐	☐	9 (10)

☐ Don't know (body_weight_vetej)   ☐ Don't know (body_length_vetej)

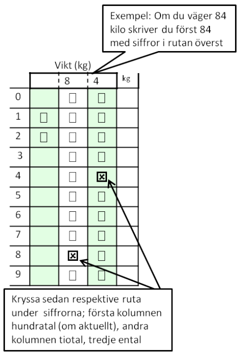

	Waist (cm)				Hip (cm)			
	(body_waist_1)	(body_waist_2)	(body_waist_3)	cm	(body_hip_1)	(body_hip_2)	(body_hip_3)	cm
0 (1)		☐	☐			☐	☐	0 (1)
1 (2)	☐	☐	☐		☐	☐	☐	1 (2)
2 (3)	☐	☐	☐		☐	☐	☐	2 (3)
3 (4)		☐	☐			☐	☐	3 (4)
4 (5)		☐	☐			☐	☐	4 (5)
5 (6)		☐	☐			☐	☐	5 (6)
6 (7)		☐	☐			☐	☐	6 (7)
7 (8)		☐	☐			☐	☐	7 (8)
8 (9)		☐	☐			☐	☐	8 (9)
9 (10)		☐	☐			☐	☐	9 (10)

☐ Don't know body_waist_vetej)   ☐ Don't know body_hip_vetej)

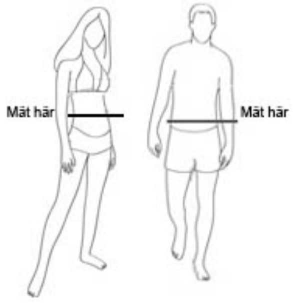

Så här mäter du ditt midjemått. Använd måttband!

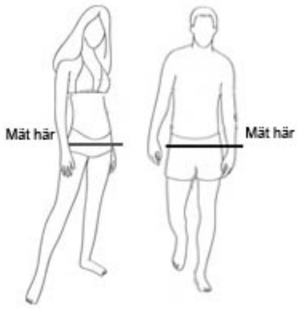

Så här mäter du ditt höftmått. Använd måttband!

10. **Do you wear glasses?** *Here we refer to corrective glasses – not lenses, sun glasses or other protective goggles.(eyeglasses)*

- ☐ No, never (0)
- ☐ Yes, sometimes (1)
- ☐ Yes, almost always (2)

11. **Are you born in Sweden?** (birth_sweden)

- ☐ Yes ⇒ *If you've ticked this alternative, jump straight to question **12*** (1)
- ☐ No ⇒ *If you've ticked this alternative, please answer the question below* (0)

When did you come to Sweden? (immigration_period)

- ☐ Before 5 years of age (1)
- ☐ When I was between 5 and 14 years old (2)
- ☐ When I was between 15 and 19 years old (3)
- ☐ When I was 20 or older (4)

12. **How many siblings do you have?** Also count half-siblings and deceased siblings. (siblings_num)

- ☐ None (0)
- ☐ 1 sibling (1)
- ☐ 2 siblings (2)
- ☐ 3 siblings (3)
- ☐ 4 siblings (4)
- ☐ 5 siblings (5)
- ☐ 6 siblings or more (6)

13. How would you characterize your father's occupation? *If you grew up mainly with a male guardian other than your biological father, consider the male person who you think have had the greatest influence on the environment you grew up in. (occupation_father)*

- ☐ Non-professional worker (1)
- ☐ Professional (2)
- ☐ Junior official (3)
- ☐ Mid-level official (4)
- ☐ Senior official (5)
- ☐ Self-employed (6)
- ☐ Farmer (7)
- ☐ Independent professional (8)
- ☐ Other (including being at home) (9)
- ☐ Don't know (999)

14. Which is your father's (or the guardian, who you considered in the last question) country of origin? (birth_region_father)

- ☐ Sweden (1)
- ☐ Other Nordic country (2)
- ☐ Other European country (3)
- ☐ Asia Minor (4)
- ☐ Far Asia (5)
- ☐ North America (6)
- ☐ South America (7)
- ☐ Africa (8)
- ☐ Australia (9)
- ☐ Other (10)
- ☐ Don't know (999)

15. **How would you characterize your mother's occupation?** *If you grew up mainly with a female guardian other than your biological mother, consider the female person who you think have had the greatest influence on the environment you grew up in*.*.(occupation_mother)*

- ☐ Non-professional worker (1)
- ☐ Professional (2)
- ☐ Junior official (3)
- ☐ Mid-level official (4)
- ☐ Senior official (5)
- ☐ Self-employed (6)
- ☐ Farmer (7)
- ☐ Independent professional (8)
- ☐ Other (including being a housewife) (9)
- ☐ Don't know (999)

16. **Which is your mother's (or the guardian, who you considered in last question) country of origin?** (birth_region_mother)

- ☐ Sweden (1)
- ☐ Other Nordic country (2)
- ☐ Other European country (3)
- ☐ Asia Minor (4)
- ☐ Far Asia (5)
- ☐ North America (6)
- ☐ South America (7)
- ☐ Africa (8)
- ☐ Australia (9)
- ☐ Other (10)
- ☐ Don't know (999)

17. **Have you daily smoked any type of tobacco during the last month?** (smoke_daily)

☐ No ⇒ *If you have ticked this alternative, jump to question* ***19*** (0)

☐ Yes (1)

18. **How much do you smoke per day?** *Tick one in each row.*

	0(0)	1-4(1)	5-9(2)	10-14(3)	15-19(4)	20-29(5)	30 or more(6)
**Cigarettes (smoke_cigarette)**	☐	☐	☐	☐	☐	☐	☐
**Cigars or cigarillos (smoke_cigar)**	☐	☐	☐	☐	☐	☐	☐
**Pipe smoking (smoke_pipe)**	☐	☐	☐	☐	☐	☐	☐

19. **Are you daily exposed to tobacco smoke from others in your home?** (smoke_secondary)

☐ No (0)

☐ Yes (1)

20. **Do you use snuff?** (snuff_amount)

☐ No (0)

☐ Yes, less than one box per week (1)

☐ Yes, 1-2 boxes per week (2)

☐ Yes, 3-7 boxes per week (3)

☐ Yes, more than 7 boxes per week (4)

21. How often do you drink..... *(tick one in each row)*

	Never (1)	Less than once a month (2)	1-3 times a month (3)	1-2 times a month (4)	3-4 times a month (5)	5-6 times a month(6)	1 every day (7)	2 times every day (8)	3 times or more per day (9)	Do not want to answer (999)
**medium beer? (alcohol_1)**	☐	☐	☐	☐	☐	☐	☐	☐	☐	☐
**strong beer? (alcohol_2)**	☐	☐	☐	☐	☐	☐	☐	☐	☐	☐
**wine? (alcohol_3)**	☐	☐	☐	☐	☐	☐	☐	☐	☐	☐
**strong wine? (alcohol_4)**	☐	☐	☐	☐	☐	☐	☐	☐	☐	☐
**liquor? (alcohol_5)**	☐	☐	☐	☐	☐	☐	☐	☐	☐	☐

With medium beer we refer to ”class II” (2,8% or 3,5%), that are for sale in ordinary food shops.

22. How much have you sunbathed outdoors on average during the last years?
Give as accurate an estimate as possible.(sun_bathe)

☐ Never (0)

☐ Less than 5 hours per year (1)

☐ 5-14 hours per year (2)

☐ 15-29 hours per year (3)

☐ 30-60 hours per year (4)

☐ More than 60 hours per year (5)

23. Do you go on holidays in the sun to warm countries?(sun_trip)

☐ Never (0)

☐ Only the occasional time (1)

☐ Each or every other year (2)

☐ Several times a year (3)

24. Do you use a sun-bed? (sun_bed)

☐ No (0)

☐ Yes, 1-10 times per year (1)

☐ Yes, 11-30 times per year (2)

☐ Yes, more than 30 times per year (3)

25. During <u>the last 4 weeks</u>, how many times did you spend 10 minutes or more in a cinema, theatre, concert- or lecture hall, café or restaurant or similar with more than for you 20 new persons? *This and the following questions refer to both work and spare time. (public_place_4weeks)*

> *NB! This question refers to premises in which you meet many, to you, <u>new</u> persons . This is why you should not count visits in work places, classrooms or other premises where you meet people that you see daily or almost daily.*

☐ None (0)

☐ Once (1)

☐ Twice (2)

☐ 3-4 times (3)

☐ 5-9 times (4)

☐ 10-19 times (5)

☐ 20 times or more (6)

26. **In total during last week, approximately how long time did you spend indoors in a closed premises with more than 20 other persons, known or unknown? (public_place_week)**

☐ 1 hour or less (1)

☐ More than 1 hour, but less than 6 hours(2)

☐ 6 hours or more, but less than 14 hours(3)

☐ 14 hours or more(4)

This question refers to premises in which you meet many persons, regardless of them being new or old aquaintances. Also count being in a cinema, theatre, concert- or lecture hall, café, or restaurant but also classroom, open-plan office , gym etc.

27. **During an ordinary weekday last week, approximately how many <u>different</u> persons were you in close contact with?** *Also count family members and contacts in your spare time, but only count <u>each individual person once</u>. Of course it is difficult to know exactly, and therefore we ask you to think carefully. Give an accurate an estimate as possible. (contact_ppl)*

*With ”close contact” we refer to having a distance between you of **<u>less than a meter</u>** for **<u>more than 1 minute</u>**.*

If you are sitting in a cinema , count the 3 persons sitting closest behind you, the person sitting closest to the right and closest to the left of you , as well as the 3 persons sitting closest in front of you, i. e. 8 persons (that is, if all seats are taken and you haven't met one or more of them earlier that day). When on public transport new persons may of course appear whilst you are sitting/standing there - then the number of contacts increases.

- ☐ 0(1)
- ☐ 1(2)
- ☐ 2-4(3)
- ☐ 5-9(4)
- ☐ 10-19 (5)
- ☐ 20-29(6)
- ☐ 30-44(7)
- ☐ 45-99(8)
- ☐ 100-149(9)
- ☐ 150 eller fler(10)

28. Of the persons you stated in question 27 that you were in close contact with ..... *(Give as accurate an estimate as possible. Tick one in each row)*

	0 (1)	1 (2)	2-4 (3)	5-9 (4)	10-19 (5)	20-29 (6)	30-44 (7)	45 or more (8)
**do you see <u>regularly</u> (at least twice a week)? (contact_ppl_rglrly)**	☐	☐	☐	☐	☐	☐	☐	☐
**were <u>children below 13 years of age</u>? (contact_ppl_under13)**	☐	☐	☐	☐	☐	☐	☐	☐
**Were you in close contact with for <u>more than 5 minutes</u>?** (contact_ppl_close)	☐	☐	☐	☐	☐	☐	☐	☐
**Did you have <u>physical contact*</u> with? (contact_ppl_physical)**	☐	☐	☐	☐	☐	☐	☐	☐

* *With physical contact we refer to <u>body contact</u>, such as for example handshakes or hugs.*

29. People have different "styles" in their interaction with others – some are more "physical" and readily touch the one they talk to, while others are more reserved and almost never touch other people other than their closest family. How would you characterize yourself? (contact_style)

☐ Physical (1)

☐ Neither physical nor reserved (2)

☐ Reserved (3)

☐ Don't know /don't want to answer (999)

30. **How often do you hug or give a kiss on the cheek to people outside your own household?** *Give as accurate an estimate as possible.(contact_hug)*

- ☐ Almost never (1)
- ☐ Sometimes, but more rarely than once a week (2)
- ☐ One or a few times per week but not every day (3)
- ☐ 1-2 times per day (4)
- ☐ 3-4 times per day (5)
- ☐ 5-9 times per day (6)
- ☐ 10 times or more per day (7)

The questionnaire is now finished. Please return it in the post-free self-addressed envelope enclosed. Thank you for your answers!
