# Supplementary material for: Cohort profile: Studies of Work Environment and Disease Epidemiology-Infections (SWEDE-I), a prospective cohort on employed adults in Sweden
Source: PLoS One. 2019 May 15;14(5):e0217012. doi: 10.1371/journal.pone.0217012 (PMC6519895; doi:10.1371/journal.pone.0217012)
Supplement: S4 File — (PDF) [file pone.0217012.s004.pdf]

4. About your health status

1. How would you rate your health status?(general_health)

- ☐ Very good (1)
- ☐ Good (2)
- ☐ Neither good nor poor (3)
- ☐ Poor (4)
- ☐ Very poor (5)

2. On average during the last 5 years, approximately how many times per year have you had colds or other upper respiratory tract infections? (sick_cold)

Also count infections that did not call for sick leave.

- ☐ None (0)
- ☐ Only the once during the whole 5-year-period (1)
- ☐ Once a year (2)
- ☐ 2 times a year (3)
- ☐ 3 times a year (4)
- ☐ 4 times a year (5)
- ☐ 5 times a year (6)
- ☐ 6 times a year or more (7)

3. On average during the last 5 years, approximately how many times per year have you suffered from stomach disease with nausea, vomiting and/or diarrhoea? (sick_stomach)

Also count infections that did not call for sick leave.

- ☐ None (1)
- ☐ Only the once during the whole 5-year-period (2)
- ☐ Once a year (3)
- ☐ 2 times a year (4)
- ☐ 3 times a year (5)
- ☐ 4 times a year (6)
- ☐ 5 times a year (7)
- ☐ 6 times a year or more (8)

4. How do you usually sleep? (sleep_quality)

- ☐ Well (1)
- ☐ Quite well (2)
- ☐ Neither well nor badly (3)
- ☐ Quite badly (4)
- ☐ Badly (5)

5. How many hours, approximately, do you usually sleep on an ordinary weekday?(sleep_duration)

- ☐ Less than 5 hours (1)
- ☐ 5 hours (2)
- ☐ 6 hours (3)
- ☐ 7 hours (4)
- ☐ 8 hours (5)
- ☐ 9 hours or more (6)

6. Do you have problems falling asleep? (sleep_trouble_falling)

- ☐ Never (1)
- ☐ Rarely (2)
- ☐ Sometimes (3)
- ☐ Most of the time (4)
- ☐ Always (5)

7. Have you been treated by a doctor for.... *(tick one in each row)*

	No (0)	Yes (1)	Don't know (999)
**Allergic skin problems (e g atopic dermatitis)? (trt_allergy_skin)**	☐	☐	☐
**Allergic problems with runny/stuffy nose (hay fever)? (trt_allergy_sneeze)**	☐	☐	☐
**Asthma? (trt_asthma)**	☐	☐	☐
**Other chronic lung disease? (trt_lung_other)**	☐	☐	☐
**Heart attack or vascular spasm? (trt_heart)**	☐	☐	☐
**High blood pressure? (trt_bloodpressure_high)**	☐	☐	☐
**Diabetes, discovered <u>before</u> the age of 30? (trt_diabetes_pre30)**	☐	☐	☐
**Diabetes, discovered <u>after</u> the age of 30? (trt_diabetes_post30)**	☐	☐	☐
**Rheumatoid arthritis? (trt_reumatism)**	☐	☐	☐
**Kidney disease? (trt_kidney)**	☐	☐	☐
**Cancer or other tumour disease?(trt_tumor)**	☐	☐	☐
**Immunodeficiency disease or reduced immune defence?** (trt_immune)	☐	☐	☐
**Undergone organ transplantation? (trt_transplant)**	☐	☐	☐

8. **Do you take any medicines regularly at the moment?** *With "regularly" we refer to the medicine being taken at least once a week and that the medication has been going on for at least 3 months and is still on-going.(med_regularly)*

☐ No, I don't take any medicine regularly ⇒ *Jump to question* ***25(0)***

☐ Yes, I take one or more medicines regularly ⇒ *Go to question* ***9(1)***

9. **Do you take any decongestants/nasal drops or nasal spray regularly at the moment?** *Example: (med_wide_nasal)*

Atrovent Nasal
Iliadin
Nasin
Nasoform
Nezeril
Noxorin
Otricomb
Otrivin
Vicks
Xylometazolin

With regularly we refer to the medicine being taken at least once a week, that the medication has been going on for at least 3 months and is still ongoing.

☐ No (0)

☐ Yes, but more rarely than once per day (1)

☐ Yes several times every day (2)

☐ Don't know (999)

10. **Do you take any of the following nasal drops or nasal spray for allergies regularly? (med_allergy_nasal)**

Lastin
Livocab
Livostin
Lomudal
Pollyferm

☐ No (0)

☐ Yes, but more rarely than once per day (1)

☐ Yes, one or several times every day (3)

☐ Don't know (999)

11. Do you take any of the following nasal drops or nasal spray with cortisone regularly? (med_cortison_nasal)

Avamys	*Desonix*	*Flutikason*	*Nasonex*
Becotide Nasal	*Flutide*	*Nasacort*	*Rhinocort*

☐ No (0)

☐ Yes, but more rarely than once per day (1)

☐ Yes, one or several times per day (2)

☐ Don't know (999)

12. Do you take any of the following decongestive inhalants or bronchodilators regularly? (med_wide_inhale)

Airomir	*Foradil*	*Oxez*	*Serevent*
Atrovent	*Formatris*	*Oxis*	*Spiriva*
Bricanyl	*Ipramol*	*Sabufarm*	*Terbasmin*
Buventol	*Ipraxa*	*Salbutamol*	*Ventilastin*
Combivent	*Onbrez*	*Sapimol*	*Ventoline*

> *With <u>regularly</u> we refer to the medicine being taken at least once a week, that the medication has been going on for at least 3 months and is still ongoing.*

☐ No (0)

☐ Yes, but more rarely than once per day (1)

☐ Yes, once or several times per day (2)

☐ Don't know (999)

13. Do you take any of the following inhalants with cortisone regularly? (med_cortison_inhale)

AeroBec
Alvesco
Asmanex
Atemur
Beclomet
Becotide
Budesonid
Flixotaide
Flixotide
Flutide
Giona
Novopulmon
Pulmicort

- ☐ No (0)
- ☐ Yes, but more rarely than once per day (1)
- ☐ Yes, one or several times per day (2)
- ☐ Don't know (999)

14. Do you take any of the following inhalants with a combination of bronchodilator/decongestive substance and cortisone regularly? (med_combo_inhale)

Assieme
Brisomax
Budfor
Edolfo
Innovair
Maizar
Rilast
Seretaide
Seretide
Sinestic
Symbicort
Veraspir

- ☐ No (0)
- ☐ Yes, but more rarely than once per day (1)
- ☐ Yes, one or several times per day (2)
- ☐ Don't know (999)

15. Do you regularly take anti-histamines (usually for allergies)? *Example: (med_antihistamines)*

Acura
Aerius
Alimemazin
Cetidura
Cetiristad
Cetrizin
Clarityn
Kestine
Lergigan
Lortadin
Nefoxef
Tavegyl
Telfast
Theralen
Zanlan
Zyrlex

- ☐ No (0)
- ☐ Yes, but more rarely than once per day (1)
- ☐ Yes, one or several times per day (2)
- ☐ Don't know (999)

16. Do you regularly take anti-inflammatory painkillers or other anti-inflammatory medicines at the moment? *Example: (med_antiinflamation)*

Acetylsalicylsyra
Albyl
Alganex
Alindrin
Alka-Selzer
Alpoxen
Alvedon
Arcoxia
Ardinex
Arthrotec
Artrox
Aspirin
Bamyl
Brexidol
Brufen
Burana
Celebra
Citodon
Comfora
Confortid
Diklofenak
Dolenio
Donacom
Eeze
Enantyum
Enaros
Eox
Glucomed
Glucosine
Glufan
Glukosamin
Ibumetin
Ibuprofen
Ipren
Ketesse
Koffazon
Magnecyl
Meloxicam
Nabumeton
Naprosyn
Naproxen
Orudis
Osaflex
Pamol
Panocod
Panodil
Paracetamol
Paracut
Perfalgan
Perigona
Piroxicam
Pronaxen
Relifex
Reliv
Siduro
Strefen
Tauxib
Tradil
Treo
Turox
Voltaren
Xefo
Xpri

☐ No (0)

☐ Yes, but more rarely than once per day (1)

☐ Yes, one or several times per day (2)

☐ Don't know (999)

17. Do you regularly take any of the following medicines for stomach ulcers, gastritis or oesophageal catarrh at the moment?(med_ulcer)

Artonil
Axagon
Caldazol
Controloc
Esopral
Famotidin
Inexium
Inside
Lanzoprazol
Lanzo
Limpidex
Losec
Nexium
Nixacid
Omeprazol
Omestom
Pantecta Control
Pantoloc
Pantoprazol
Pantozol
Pariet
Pepcid
Pepcid Duo
Rani-Q
Ranitidin
Somac
Zantac
Zapanzol
Zindazol
Zoton

☐ No (0)

☐ Yes, but more rarely than once per day (1)

☐ Yes, one or several times per day (2)

☐ Don't know (999)

18. Do you at the moment regularly take any of the following medicines for stomach problems: Egazil, Propantelin? (med_egazil)

- [ ] No (0)
- [ ] Yes, but more rarely than once per day (1)
- [ ] Yes, one or several times per day (2)
- [ ] Don't know (999)

19. Do you take insulin syringes at the moment? (med_insulin)

- [ ] No (0)
- [ ] Yes (1)
- [ ] Don't know (999)

20. Do you at the moment regularly take any other medicine for diabetes? (med_diabetes_other)

- [ ] No (0)
- [ ] Yes (1)
- [ ] Don't know (999)

21. Do you at the moment regularly take any cortisone tablets? *Example: (med_kortison_pill)*

Betapred
Deltison
Dexacortal
Dexametason
Hydrocortison
Kortisonacetat
Lodotra
Medrol
Prednisolon

- [ ] No (0)
- [ ] Yes (1)
- [ ] Don't know (999)

22. **Do you at the moment regularly take any blood lipid-reducing medicines?** *Example:* **(med_bloodfat)**

Atorvastatin	*Pravachol*	*Tahor*	*Vasta*
Crestor	*Pravastatin*	*Torvarin*	*Zarator*
Lescol	*Simvastatin*	*Torvast*	*Zocord*
Lipitor	*Sortis*	*Vabadin*	

☐ No (0)

☐ Yes (1)

☐ Don't know (999)

23. **Do you at the moment regularly take any cardiac medicines? (med_heart)**

☐ No (0)

☐ Yes (1)

☐ Don't know (999)

24. **Do you at the moment regularly take anti-hypertensive agents? (med_bloodpressure)**

☐ No (0)

☐ Yes (1)

☐ Don't know (999)

25. **Do you take any medicines seasonally?** *With "seasonally" we refer to the medicine being taken at least once a week during a part of the year, but maybe not right now.(med_seasonal)*

☐ No, I don't take any medicines seasonally ⇒ *Jump to question* ***33(0)***

☐ Yes, I take one or more medicines seasonally ⇒ *Go to question* ***26(1)***

26. Do you take any decongestants/nasal drops or nasal spray seasonally?
Example: (med_wide_seasonal)

Atrovent Nasal	*Nasoform*	*Otricomb*	*Xylometazolin*
Iliadin	*Nezeril*	*Otrivin*	
Nasin	*Noxorin*	*Vicks*	

With seasonally we refer to the medicine being taken at least once a week for part of the year, but maybe not right now

☐ No (0)

☐ Yes (1)

☐ Don’t know (999)

27. Do you take any of the following decongestants/nasal drops or nasal spray for allergies seasonally: Lastin, Livocab, Livostin, Lomudal, Pollyferm? (med_allergy_nasal_seas)

☐ No (0)

☐ Yes (1)

☐ Don’t know (999)

28. Do you take any decongestants/nasal drops or nasal spray with cortisone seasonally? (med_cortison_nasal_seas)

Avamys	*Desonix*	*Flutikason*	*Nasonex*
Becotide Nasal	*Flutide*	*Nasacort*	*Rhinocort*

☐ No (0)

☐ Yes (1)

☐ Don’t know (999)

29. Do you seasonally take any of the following decongestive inhalators or bronchodilators? (med_wide_inhale_seas)

Airomir	*Foradil*	*Oxez*	*Serevent*
Atrovent	*Formatris*	*Oxis*	*Spiriva*
Bricanyl	*Ipramol*	*Sabufarm*	*Terbasmin*
Buventol	*Ipraxa*	*Salbutamol*	*Ventilastin*
Combivent	*Onbrez*	*Sapimol*	*Ventoline*

☐ No (0)

☐ Yes (1)

☐ Don't know (999)

30. Do you seasonally take any of the following inhalators with cortisone? (med_cortison_inhale_seas)

AeroBec	*Beclomet*	*Flixotaide*	*Giona*
Alvesco	*Becotide*	*Flixotide*	*Novopulmon*
Asmanex	*Budesonid*	*Flutide*	*Pulmicort*
Atemur			

☐ No (0)

☐ Yes (1)

☐ Don't know (999)

31. Do you seasonally take any of the following inhalators with a combination of bronchodilators/decongestive substance and cortisone? (med_combo_inhale_seas)

Assieme	*Edolfo*	*Rilast*	*Sinestic*
Brisomax	*Innovair*	*Seretaide*	*Symbicort*
Budfor	*Maizar*	*Seretide*	*Veraspir*

☐ No (0)

☐ Yes (1)

☐ Don't know (999)

32. **Do you seasonally take anti-histamines (usually for allergies)?** *Example:* *(med_antihist_seas)*

Acura	*Cetiristad*	*Lergigan*	*Telfast*
Aerius	*Cetrizin*	*Lortadin*	*Theralen*
Alimemazin	*Clarityn*	*Nefoxef*	*Zanlan*
Cetidura	*Kestine*	*Tavegyl*	*Zyrlex*

☐ No (0)

☐ Yes (1)

☐ Don’t know (999)

33. **Do you ever take any herbal remedies for cold, i e remedies that can be bought for instance in health food stores, such as Kan Yang, Echinacea, Echinaforte, Echinagard, Esberitox, Sinova?** *Give as accurate an estimate as possible.* (med_plant_cold)

With ”time” we refer to one course of treatment or round, that may consist of one or more doses.

☐ Never (0)

☐ 1-4 times a year (1)

☐ 5-9 times a year (2)

☐ 10-19 times a year (3)

☐ 20 times or more per year (4)

☐ Don’t know (5)

34. Do you ever take Vitamin C in order to prevent colds? Give as accurate an estimate as possible. (med_vtmn_C)

With "time" we refer to one course of treatment or round, that may consist of one or more doses.

- ☐ Never (0)
- ☐ 1-4 times a year (1)
- ☐ 5-9 times a year (2)
- ☐ 10-19 times a year (3)
- ☐ 20 times or more per year (4)
- ☐ Don't know (999)

35. Did you get a vaccination against the new flu ("swine flu") in 2009 or 2010? (vacc_flu_swine)

As you may remember, a special flu vaccin against the new flu (the "swine flu") was offered to the public in connection with the flu epidemic . <u>This question</u> refers to that actual vaccination. From the autumn of 2010 there is a protection against the new flu in the vaccin against the yearly flu, but this question does <u>not</u> refer to that vaccination (that started in October 2010).

- ☐ Yes, one injection (1)
- ☐ Yes, two injections *(2)*
- ☐ No (3)
- ☐ Don't know (999)

36. **Did you get a vaccination against the yearly flu at any time during the period October 2010 – September 2011? (vacc_flu_yearly)**

- ☐ Yes (1)
- ☐ No (2)
- ☐ Don't know (999)

37. **During the last year, at how many times in total have you been at work whilst you have felt that you have an on-going infection in your body?** *Give as accurate an estimate as possible. (infection_at_work_with)*

- ☐ None (1)
- ☐ Once (2)
- ☐ 2-3 times (3)
- ☐ 4-5 times (4)
- ☐ More than 5 times (5)

38. **During the last year, at approximately how many times have you visited a website with medical information (i e 1177.se) when you have had colds or other upper respiratory infections? (infection_research)**

- ☐ None (0)
- ☐ Once (1)
- ☐ 2 times (2)
- ☐ 3 times (3)
- ☐ 4 times (4)
- ☐ 5 times (5)
- ☐ 6 times (6)
- ☐ 7 times or more (7)

39. **When knowing that many people at your work place are ill, do you do anything to avoid getting infected?** *Tick all the alternatives that match how you usually do.*

- ☐ Yes, I avoid people who seem to be ill (imeas_avoid_sick)
- ☐ Yes, I try to avoid as many personal contacts I can, also with healthy persons (imeas_avoid_all)
- ☐ Yes, I travel to work in other ways than I usually do (imeas_different_transp)
- ☐ Yes, I work from home (imeas_work_at_home)
- ☐ Yes, when talking to colleagues I avoid being close to them (imeas_keep_distance)
- ☐ Yes, I avoid touching door knobs and similar things that many people touch (imeas_avoid_knobs)
- ☐ Yes, I wash many hands more often and/or more efficiently than I usually do (imeas_wash_hands)
- ☐ Yes, I use disinfectant more often than I usually do (imeas_desinfectant)
- ☐ Yes, I avoid touching my face, mouth and eyes (imeas_avoid_own_face)
- ☐ Yes, I buy over-the-counter medicines or herbal cold remedies (imeas_alt_medicine)
- ☐ Yes, I take other measures than the ones mentioned above (imeas_other)
- ☐ No, I don't take any measures (imeas_no_measures)
- ☐ No, I cannot remember there ever being any infective disease at my place of work (imeas_no_infection)

The questionnaire is now finished. Please return it in the post-free self-addressed envelope enclosed. Thank you for your answers!
