# Supplementary material for: Cohort profile: Studies of Work Environment and Disease Epidemiology-Infections (SWEDE-I), a prospective cohort on employed adults in Sweden
Source: PLoS One. 2019 May 15;14(5):e0217012. doi: 10.1371/journal.pone.0217012 (PMC6519895; doi:10.1371/journal.pone.0217012)
Supplement: S5 File — (PDF) [file pone.0217012.s005.pdf]

Sara Christensen, 2012-06-27

Questionnaire 5

Physical activity and dietary habits

The SWEDE-I study 2011-2012

Physical activity

On next page the section on physical activity starts

q715 *(PHY_OCCAT_1)*

What is your normal level of activity at work?

	Mostly sitting<br><br>1 (1)	2<br>(2)	Standing or walking most of the time<br><br>3 (3)	4<br>(4)	Heavy labor<br><br>5 (5)	Don't know (998)	Don't want to answer (999)
(1)	❍	❍	❍	❍	❍	❍	❍

q404 *(PHY3_OCCAT_DUR)*

How long is your normal working day?

❍ 30 minutes (1)
❍ 1 hour (2)
❍ 1 hour and 30 minutes (3)
❍ 2 hours (4)
❍ 2 hours and 30 minutes (5)
❍ 3 hours (6)
❍ 3 hours and 30 minutes (7)
❍ 4 hours (8)
❍ 4 hours and 30 minutes (9)
❍ 5 hours (10)
❍ 5 hours and 30 minutes (11)
❍ 6 hours (12)
❍ 6 hours and 30 minutes (13)
❍ 7 hours (14)
❍ 7 hours and 30 minutes (15)
❍ 8 hours (16)
❍ 8 hours 30 minutes (17)

❍ 9 hours (18)
❍ 9 hours and 30 minutes (19)
❍ 10 hours (20)
❍ 10 hours and 30 minutes (21)
❍ 11 hours (22)
❍ 11 hours and 30 minutes (23)
❍ 12 hours (24)
❍ Don't know (998) [Keep position ◆ Exclusive]
❍ Don't want to answer (999) [Keep position ◆ Exclusive]

g397

You stated how long your normal working day is.

How many hours and minutes of your normal working day do you engage in activities that require the effort similar to...

	q395 - Hours	q396 - Minutes
A. For example, sleep or rest (1) *(Q395_1 PHY_ACT_SLEEP_HOURS) (Q396_1 PHY_ACT_SLEEP_MIN)*	❍ - (0)<br>❍ 0 (00)<br>❍ 1 hour (1)<br>❍ 2 hours (2)<br>❍ 3 hours (3)<br>❍ 4 hours (4)<br>❍ 5 hours (5)<br>❍ 6 hours (6)<br>❍ 7 hours (7)<br>❍ 8 hours (8)<br>❍ 9 hours (9)<br>❍ 10 hours (10)<br>❍ 11 hours (11)<br>❍ 12 hours (12)	❍ - (0)<br>❍ 0 (00)<br>❍ 5 minutes (1)<br>❍ 10 minutes (2)<br>❍ 15 minutes (3)<br>❍ 20 minutes (4)<br>❍ 25 minutes (5)<br>❍ 30 minutes (6)<br>❍ 35 minutes (7)<br>❍ 40 minutes (8)<br>❍ 45 minutes (9)<br>❍ 50 minutes (10)<br>❍ 55 minutes (11)
B. For example, sitting in the bath tub, sitting and listening to music, watching TV (2) *(Q395_2 PHY_ACT_TV_HOURS) (Q396_2 PHY_ACT_TV_MIN)*	❍ - (0)<br>❍ 0 (00)<br>❍ 1 hour (1)<br>❍ 2 hours (2)<br>❍ 3 hours (3)<br>❍ 4 hours (4)<br>❍ 5 hours (5)<br>❍ 6 hours (6)<br>❍ 7 hours (7)<br>❍ 8 hours (8)<br>❍ 9 hours (9)<br>❍ 10 hours (10)<br>❍ 11 hours (11)<br>❍ 12 hours (12)	❍ - (0)<br>❍ 0 (00)<br>❍ 5 minutes (1)<br>❍ 10 minutes (2)<br>❍ 15 minutes (3)<br>❍ 20 minutes (4)<br>❍ 25 minutes (5)<br>❍ 30 minutes (6)<br>❍ 35 minutes (7)<br>❍ 40 minutes (8)<br>❍ 45 minutes (9)<br>❍ 50 minutes (10)<br>❍ 55 minutes (11)
C. For example, office work, knitting, sewing,	❍ - (0)	❍ - (0)

	q395 - Hours	q396 - Minutes
sitting at a meeting (3) *(Q395_3 PHY_ACT_OFFWORK_HOURS) (Q396_3 PHY_ACT_OFFWORK_MIN)*	❍ 0 (00)<br>❍ 1 hour (1)<br>❍ 2 hours (2)<br>❍ 3 hours (3)<br>❍ 4 hours (4)<br>❍ 5 hours (5)<br>❍ 6 hours (6)<br>❍ 7 hours (7)<br>❍ 8 hours (8)<br>❍ 9 hours (9)<br>❍ 10 hours (10)<br>❍ 11 hours (11)<br>❍ 12 hours (12)	❍ 0 (00)<br>❍ 5 minutes (1)<br>❍ 10 minutes (2)<br>❍ 15 minutes (3)<br>❍ 20 minutes (4)<br>❍ 25 minutes (5)<br>❍ 30 minutes (6)<br>❍ 35 minutes (7)<br>❍ 40 minutes (8)<br>❍ 45 minutes (9)<br>❍ 50 minutes (10)<br>❍ 55 minutes (11)
D. For example, making the bed, ironing clothes, washing up by hand (4) *(Q395_4 PHY_ACT_IRONING_HOURS) (Q396_4 PHY_ACT_IRONING_MIN)*	❍ - (0)<br>❍ 0 (00)<br>❍ 1 hour (1)<br>❍ 2 hours (2)<br>❍ 3 hours (3)<br>❍ 4 hours (4)<br>❍ 5 hours (5)<br>❍ 6 hours (6)<br>❍ 7 hours (7)<br>❍ 8 hours (8)<br>❍ 9 hours (9)<br>❍ 10 hours (10)<br>❍ 11 hours (11)<br>❍ 12 hours (12)	❍ - (0)<br>❍ 0 (00)<br>❍ 5 minutes (1)<br>❍ 10 minutes (2)<br>❍ 15 minutes (3)<br>❍ 20 minutes (4)<br>❍ 25 minutes (5)<br>❍ 30 minutes (6)<br>❍ 35 minutes (7)<br>❍ 40 minutes (8)<br>❍ 45 minutes (9)<br>❍ 50 minutes (10)<br>❍ 55 minutes (11)
E. For example, bowling, driving a bus/tractor, shop working, working with the car, dancing waltz/foxtrot (5) *(Q395_5 PHY_ACT_BOWLING_HOURS) (Q396_5 PHY_ACT_BOWLING_MIN)*	❍ - (0)<br>❍ 0 (00)<br>❍ 1 hour (1)<br>❍ 2 hours (2)<br>❍ 3 hours (3)<br>❍ 4 hours (4)<br>❍ 5 hours (5)<br>❍ 6 hours (6)<br>❍ 7 hours (7)<br>❍ 8 hours (8)<br>❍ 9 hours (9)<br>❍ 10 hours (10)<br>❍ 11 hours (11)<br>❍ 12 hours (12)	❍ - (0)<br>❍ 0 (00)<br>❍ 5 minutes (1)<br>❍ 10 minutes (2)<br>❍ 15 minutes (3)<br>❍ 20 minutes (4)<br>❍ 25 minutes (5)<br>❍ 30 minutes (6)<br>❍ 35 minutes (7)<br>❍ 40 minutes (8)<br>❍ 45 minutes (9)<br>❍ 50 minutes (10)<br>❍ 55 minutes (11)
F. For example, taking a brisk walk, horse riding, sweeping the pavement (6) *(Q395_6*	❍ - (0)<br>❍ 0 (00)	❍ - (0)<br>❍ 0 (00)

	q395 - Hours	q396 - Minutes
PHY_ACT_WALK_HOURS) (Q396_6 PHY_ACT_WALK_MIN)	❍ 1 hour (1)<br>❍ 2 hours (2)<br>❍ 3 hours (3)<br>❍ 4 hours (4)<br>❍ 5 hours (5)<br>❍ 6 hours (6)<br>❍ 7 hours (7)<br>❍ 8 hours (8)<br>❍ 9 hours (9)<br>❍ 10 hours (10)<br>❍ 11 hours (11)<br>❍ 12 hours (12)	❍ 5 minutes (1)<br>❍ 10 minutes (2)<br>❍ 15 minutes (3)<br>❍ 20 minutes (4)<br>❍ 25 minutes (5)<br>❍ 30 minutes (6)<br>❍ 35 minutes (7)<br>❍ 40 minutes (8)<br>❍ 45 minutes (9)<br>❍ 50 minutes (10)<br>❍ 55 minutes (11)
G. For example, painting the house, carrying and stacking firewood cross country skiing/slalom skiing(7) *(Q395_7 PHY_ACT_PAINTHOUSE_HOURS) (Q396_7 PHY_ACT_PAINTHOUSE_MIN)*	❍ - (0)<br>❍ 0 (00)<br>❍ 1 hour (1)<br>❍ 2 hours (2)<br>❍ 3 hours (3)<br>❍ 4 hours (4)<br>❍ 5 hours (5)<br>❍ 6 hours (6)<br>❍ 7 hours (7)<br>❍ 8 hours (8)<br>❍ 9 hours (9)<br>❍ 10 hours (10)<br>❍ 11 hours (11)<br>❍ 12 hours (12)	❍ - (0)<br>❍ 0 (00)<br>❍ 5 minutes (1)<br>❍ 10 minutes (2)<br>❍ 15 minutes (3)<br>❍ 20 minutes (4)<br>❍ 25 minutes (5)<br>❍ 30 minutes (6)<br>❍ 35 minutes (7)<br>❍ 40 minutes (8)<br>❍ 45 minutes (9)<br>❍ 50 minutes (10)<br>❍ 55 minutes (11)
H. For example, road construction, manually mowing the lawn, sweeping snow (8) *(Q395_8 PHY_ACT_ROAD_HOURS) (Q396_8 PHY_ACT_ROAD_MIN)*	❍ - (0)<br>❍ 0 (00)<br>❍ 1 hour (1)<br>❍ 2 hours (2)<br>❍ 3 hours (3)<br>❍ 4 hours (4)<br>❍ 5 hours (5)<br>❍ 6 hours (6)<br>❍ 7 hours (7)<br>❍ 8 hours (8)<br>❍ 9 hours (9)<br>❍ 10 hours (10)<br>❍ 11 hours (11)<br>❍ 12 hours (12)	❍ - (0)<br>❍ 0 (00)<br>❍ 5 minutes (1)<br>❍ 10 minutes (2)<br>❍ 15 minutes (3)<br>❍ 20 minutes (4)<br>❍ 25 minutes (5)<br>❍ 30 minutes (6)<br>❍ 35 minutes (7)<br>❍ 40 minutes (8)<br>❍ 45 minutes (9)<br>❍ 50 minutes (10)<br>❍ 55 minutes (11)
How many hours per day do you pursue activities that require more effort than alternative H? (9) *(Q395_9*	❍ - (0)<br>❍ 0 (00)<br>❍ 1 hour (1)	❍ - (0)<br>❍ 0 (00)<br>❍ 5 minutes (1)

	q395 - Hours	q396 - Minutes
PHY_ACT_MORE_HOURS) (Q396_9 PHY_ACT_MORE_MIN)	❍ 2 hours (2)<br>❍ 3 hours (3)<br>❍ 4 hours (4)<br>❍ 5 hours (5)<br>❍ 6 hours (6)<br>❍ 7 hours (7)<br>❍ 8 hours (8)<br>❍ 9 hours (9)<br>❍ 10 hours (10)<br>❍ 11 hours (11)<br>❍ 12 hours (12)	❍ 10 minutes (2)<br>❍ 15 minutes (3)<br>❍ 20 minutes (4)<br>❍ 25 minutes (5)<br>❍ 30 minutes (6)<br>❍ 35 minutes (7)<br>❍ 40 minutes (8)<br>❍ 45 minutes (9)<br>❍ 50 minutes (10)<br>❍ 55 minutes (11)

Make sure that the sum is equivalent to the length of your normal working day.

q869 *(Q869_1 PHY_SPARTIME)*

In your spare time, how physically active would you say that you are usually?

	Mostly sitting 1 (1)	2 (2)	Walking 30 minutes per day 3 (3)	4 (4)	Performing a challenging activity 60 minutes per day 5 (5)	Don't know (998)	Don't want to answer (999)
(1)	❍	❍	❍	❍	❍	❍	❍

q722

Which of the following activities do you pursue at least once a week, during your spare time?

❑ Sitting and watching TV, DVD etc. (2) *(Q722_2 PHY3_LEIACT_TV)*
❑ Sitting by the computer, reading e-mail, playing PC-games, play station, Xbox (3) *(Q722_3 PHY3_LEIACT_COM)*
❑ Sitting and reading, writing, sewing etc. (4) *(Q722_4 PHY3_LEIACT_REA)*
❑ Going out dancing, e g disco or country-dancing (9) *(Q722_9 PHY3_LEIACT_DAN)*
❑ Walking (not as a means of transport to daily occupation), Nordic walking, walking a dog (1001) *(Q722_1001 PHY3_LEIACT_WAL)*

❑ Playing an instrument or computer- and TV games that require physical activity, e g Wii (5) (Q722_5 PHY3_LEIACT_PHY)
❑ Household work, cleaning, doing the laundry, taking care of children, gardening work etc. (6) (Q722_6 PHY3_LEIACT_HOM)
❑ Shopping or running errands (7) (Q722_7 PHY3_LEIACT_SHO)
❑ Riding a bike (not as means of transport to daily occupation) (1101) *(Q722_1101 PHY3_LEIACT_BIC)*
❑ Other (h1)
❍ None of these (0) *(Q722_0 PHY3_LEIACT_0)*
❍ Don't know (998) *(Q722_998 PHY3_LEIACT_998)*
❍ Don't want to answer (999) *(Q722_999 PHY3_LEIACT_999)*

g725

How often do you dedicate your time to the following recreational activities? And for how long time every day?

	Number of days a week	Total time per day
Sitting and watching TV, DVD etc. *(Q723_2 PHY3_LEIACT1_TV_FRQ) (Q724_2 PHY3_LEIACT1_TV_DUR)*	❍ 1 (1)<br>❍ 2 (2)<br>❍ 3 (3)<br>❍ 4 (4)<br>❍ 5 (5)<br>❍ 6 (6)<br>❍ 7 (7)<br>❍ Don't know (998)<br>❍ Don't want to answer (999)	❍ Less than 30 minutes (1)<br>❍ 30 – 59 minutes (2)<br>❍ 60 - 119 minutes (3)<br>❍ 2 - 4 hours (4)<br>❍ 5 - 8 hours (5)<br>❍ More than 8 hours (6)<br>❍ Don't know (998)<br>❍ Don't want to answer (999)
Sitting by the computer, reading emails, playing PC games, play station, Xbox etc. *(Q723_3 PHY3_LEIACT1_COM_FRQ)(Q724_3 PHY3_LEIACT1_COM_DUR)*	❍ 1 (1)<br>❍ 2 (2)<br>❍ 3 (3)<br>❍ 4 (4)<br>❍ 5 (5)<br>❍ 6 (6)<br>❍ 7 (7)<br>❍ Don't know (998)<br>❍ Don't want to answer (999)	❍ Less than 30 minutes (1)<br>❍ 30 – 59 minutes (2)<br>❍ 60 - 119 minutes (3)<br>❍ 2 - 4 hours (4)<br>❍ 5 - 8 hours (5)<br>❍ More than 8 hours (6)<br>❍ Don't know (998)<br>❍ Don't want to answer (999)
Sitting and reading, writing, sewing etc. *(Q723_4 PHY3_LEIACT1_REA_FRQ) (Q724_4 PHY3_LEIACT1_REA_DUR)*	❍ 1 (1)<br>❍ 2 (2)<br>❍ 3 (3)<br>❍ 4 (4)<br>❍ 5 (5)<br>❍ 6 (6)<br>❍ 7 (7)<br>❍ Don't know (998)<br>❍ Don't want to answer (999)	❍ Less than 30 minutes (1)<br>❍ 30 – 59 minutes (2)<br>❍ 60 - 119 minutes (3)<br>❍ 2 - 4 hours (4)<br>❍ 5 - 8 hours (5)<br>❍ More than 8 hours (6)<br>❍ Don't know (998)<br>❍ Don't want to answer (999)
Playing an instrument or computer- and TV games that require some physical activity, ex Wii *(Q723_5*	❍ 1 (1)<br>❍ 2 (2)<br>❍ 3 (3)<br>❍ 4 (4)<br>❍ 5 (5)	❍ Less than 30 minutes (1)<br>❍ 30 – 59 minutes (2)<br>❍ 60 - 119 minutes (3)<br>❍ 2 - 4 hours (4)<br>❍ 5 - 8 hours (5)

	Number of days a week	Total time per day
PHY3_LEIACT1_PHY_FRQ)(Q724_5 PHY3_LEIACT1_PHY_DUR)	❍ 6 (6)<br>❍ 7 (7)<br>❍ Don't know (998)<br>❍ Don't want to answer (999)	❍ More than 8 hours (6)<br>❍ Don't know (998)<br>❍ Don't want to answer (999)
Household work, cleaning, doing the laundry, taking care of children, gardening work etc. *(Q723_6 PHY3_LEIACT1_HOM_FRQ) (Q724_6 PHY3_LEIACT1_HOM_DUR)*	❍ 1 (1)<br>❍ 2 (2)<br>❍ 3 (3)<br>❍ 4 (4)<br>❍ 5 (5)<br>❍ 6 (6)<br>❍ 7 (7)<br>❍ Don't know (998)<br>❍ Don't want to answer (999)	❍ Less than 30 minutes (1)<br>❍ 30 – 59 minutes (2)<br>❍ 60 - 119 minutes (3)<br>❍ 2 - 4 hours (4)<br>❍ 5 - 8 hours (5)<br>❍ More than 8 hours (6)<br>❍ Don't know (998)<br>❍ Don't want to answer (999)
Shopping or running errands *(Q723_7 PHY3_LEIACT1_SHO_FRQ) (Q724_7 PHY3_LEIACT1_SHO_DUR)*	❍ 1 (1)<br>❍ 2 (2)<br>❍ 3 (3)<br>❍ 4 (4)<br>❍ 5 (5)<br>❍ 6 (6)<br>❍ 7 (7)<br>❍ Don't know (998)<br>❍ Don't want to answer (999)	❍ Less than 30 minutes (1)<br>❍ 30 – 59 minutes (2)<br>❍ 60 - 119 minutes (3)<br>❍ 2 - 4 hours (4)<br>❍ 5 - 8 hours (5)<br>❍ More than 8 hours (6)<br>❍ Don't know (998)<br>❍ Don't want to answer (999)
Going out dancing for example disco or country-dance *(Q724_9 PHY3_LEIACT1_DAN_DUR) (Q724_9 PHY3_LEIACT1_DAN_DUR)*	❍ 1 (1)<br>❍ 2 (2)<br>❍ 3 (3)<br>❍ 4 (4)<br>❍ 5 (5)<br>❍ 6 (6)<br>❍ 7 (7)<br>❍ Don't know (998)<br>❍ Don't want to answer (999)	❍ Less than 30 minutes (1)<br>❍ 30 – 59 minutes (2)<br>❍ 60 - 119 minutes (3)<br>❍ 2 - 4 hours (4)<br>❍ 5 - 8 hours (5)<br>❍ More than 8 hours (6)<br>❍ Don't know (998)<br>❍ Don't want to answer (999)

g875

Walking (not as a means of transport to daily occupation), Nordic walking, walking a dog *(Q873_1001 PHY3_LEIACT2_WAL_FRQ) (Q874_1001 PHY3_LEIACT2_WAL_DUR)*	❍ 1 (1)<br>❍ 2 (2)<br>❍ 3 (3)<br>❍ 4 (4)<br>❍ 5 (5)<br>❍ 6 (6)<br>❍ 7 (7)<br>❍ Don't know (998)<br>❍ Don't want to answer (999)	❍ Less than 15 minutes (1)<br>❍ 15 –29 minutes (2)<br>❍ 30 - 44 minutes (3)<br>❍ 45 - 59 minutes (4)<br>❍ 1 - 2 hours (5)<br>❍ More than 2 hours (6)<br>❍ Don't know (998)<br>❍ Don't want to answer (999)
Riding a bike (not as a means of transport to daily occupation)	❍ 1 (1)<br>❍ 2 (2)<br>❍ 3 (3)	❍ Less than 15 minutes (1)<br>❍ 15 –29 minutes (2)<br>❍ 30 - 44 minutes (3)

(Q873_1101 PHY3_LEIACT2_BIC_FRQ) (Q874_1101 PHY3_LEIACT2_BIC_DUR)	❍ 4 (4)<br>❍ 5 (5)<br>❍ 6 (6)<br>❍ 7 (7)<br>❍ Don't know (998)<br>❍ Don't want to answer (999)	❍ 45 - 59 minutes (4)<br>❍ 1 - 2 hours (5)<br>❍ More than 2 hours (6)<br>❍ Don't know (998)<br>❍ Don't want to answer (999)

q728 *(PHY2_SPORT)*

Do you exercise or practice any kind of sports regularly?

❍ Yes (1) [Exclusive]
❍ No (0) [Exclusive]
❍ Don't know (998) [Keep position ◆ Exclusive]
❍ Don't want to answer (999) [Keep position ◆ Exclusive]

q729

What type of exercise or sports activity do you normally practice?

If your type of activity is not included in the list below, please pick a similar one.

❑ Gymnastics or aerobics (1) *(Q729_1 PHY3_SPORTS_AER)*
❑ Weight training (2) *(Q729_2 PHY3_SPORTS_GYM)*
❑ Jogging, running or orienteering (3) *(Q729_3 PHY3_SPORTS_JOG)*
❑ Athletics, for example high jump, long jump or triple jump (4) *(Q729_4 PHY3_SPORTS_ATH)*
❑ Spinning or cycling in rough terrains (5) *(Q729_5 PHY3_SPORTS_SPI)*
❑ Swimming (6) *(Q729_6 PHY3_SPORTS_SWI)*
❑ Team ball games, for example football, basketball, volley ball or floor ball (7) *(Q729_7*
❑ Dance course or competitive dancing e g couple dancing, ballet, jazz or street dance(9) *(Q729_9) PHY3_SPORTS_DAN)*
❑ Horseback riding (10) *(Q729_10 PHY3_SPORTS_HOR)*
❑ Ice-skating, hockey or bandy (11) *(Q729_11 PHY3_SPORTS_HOC)*
❑ Skiing, downhill or cross country (12) *(Q729_12 PHY3_SPORTS_SKI)*
❑ Martial arts, e g Judo or Karate (13) *(Q729_13 PHY3_SPORTS_MAR)*
❑ Boxing or wrestling (14) *(Q729_14 PHY3_SPORTS_BOX)*
❑ Yoga, Pilates or Tai chi (15) *(Q729_15*
❑ Sailing, surfing, canoeing or rowing (17) *(Q729_17 PHY3_SPORTS_SAI)*
❑ Motorsports, e g motocross (18) *(Q729_18 PHY3_SPORTS_MOT)*
❑ Climbing (19) *(Q729_19 PHY3_SPORTS_CLI)*
❑ Other (h1)
❍ None of these (0) *(Q729_0 PHY3_SPORTS_0)*
❍ Don't now (998) *(Q729_998 PHY3_SPORTS_998)*
❍ Don't want to answer (999) *(Q729_999 PHY3_SPORTS_999)*

PHY3_SPORTS_BAL)
❑ Golf (8) *(Q729_8 PHY3_SPORTS_GOL)*
PHY3_SPORTS_YOG)
❑ Tennis, badminton or table tennis (20) *(Q729_20 PHY3_SPORTS_TEN)*
❑ Squash (21) *(Q729_21 PHY3_SPORTS_SQU)*

q732

How often do you dedicate yourself to the following sports and for how long each time?

	Number of times	Time per session
Gymnastics or aerobics *(Q730_1 PHY3_SPORTS_AER_FRQ) (Q731_1 PHY3_SPORTS_AER_DUR)*	❍ 1 - 3 times per month (1)<br>❍ 1 time per week (2)<br>❍ 2 - 3 times per week (3)<br>❍ 4 - 5 times per week (4)<br>❍ 6 - 7 times per week (5)<br>❍ Don't now (998)<br>❍ Don't want to answer (999)	❍ Less than 30 minutes<br>❍ 30 - 59 minutes<br>❍ 60 - 119 minutes<br>❍ 2 - 4 hours<br>❍ More than 4 hours<br>❍ Don't now (998)<br>❍ Don't want to answer (999)
Weight training *(Q730_2 PHY3_SPORTS_GYM_FRQ) (Q731_2 PHY3_SPORTS_GYM_DUR)*	❍ 1 - 3 times per month (1)<br>❍ 1 time per week (2)<br>❍ 2 - 3 times per week (3)<br>❍ 4 - 5 times per week (4)<br>❍ 6 - 7 times per week (5)<br>❍ Don't now (998)<br>❍ Don't want to answer (999)	❍ Less than 30 minutes<br>❍ 30 - 59 minutes<br>❍ 60 - 119 minutes<br>❍ 2 - 4 hours<br>❍ More than 4 hours<br>❍ Don't now (998)<br>❍ Don't want to answer (999)
Jogging, running or orienteering *(Q730_3 PHY3_SPORTS_JOG_FRQ) (Q731_3 PHY3_SPORTS_JOG_DUR)*	❍ 1 - 3 times per month (1)<br>❍ 1 time per week (2)<br>❍ 2 - 3 times per week (3)<br>❍ 4 - 5 times per week (4)<br>❍ 6 - 7 times per week (5)<br>❍ Don't now (998)<br>❍ Don't want to answer (999)	❍ Less than 30 minutes<br>❍ 30 - 59 minutes<br>❍ 60 - 119 minutes<br>❍ 2 - 4 hours<br>❍ More than 4 hours<br>❍ Don't now (998)<br>❍ Don't want to answer (999)
Athletics, e g high jump, long jump or triple jump *(Q730_4 PHY3_SPORTS_ATH_FRQ) (Q731_4 PHY3_SPORTS_ATH_DUR)*	❍ 1 - 3 times per month (1)<br>❍ 1 time per week (2)<br>❍ 2 - 3 times per week (3)<br>❍ 4 - 5 times per week (4)<br>❍ 6 - 7 times per week (5)<br>❍ Don't now (998)<br>❍ Don't want to answer (999)	❍ Less than 30 minutes<br>❍ 30 - 59 minutes<br>❍ 60 - 119 minutes<br>❍ 2 - 4 hours<br>❍ More than 4 hours<br>❍ Don't now (998)<br>❍ Don't want to answer (999)
Spinning or cycling in rough *terrains (Q730_5 PHY3_SPORTS_SPI_FRQ)*	❍ 1 - 3 times per month (1)	❍ Less than 30 minutes<br>❍ 30 - 59 minutes

	Number of times	Time per session
(Q731_5 PHY3_SPORTS_SPI_DUR)	❍ 1 time per week (2)<br>❍ 2 - 3 times per week (3)<br>❍ 4 - 5 times per week (4)<br>❍ 6 - 7 times per week (5)<br>❍ Don't now (998)<br>❍ Don't want to answer (999)	❍ 60 - 119 minutes<br>❍ 2 - 4 hours<br>❍ More than 4 hours<br>❍ Don't now (998)<br>❍ Don't want to answer (999)
Swimming *(Q730_6 PHY3_SPORTS_SWI_FRQ) (Q731_6 PHY3_SPORTS_SWI_DUR)*	❍ 1 - 3 times per month (1)<br>❍ 1 time per week (2)<br>❍ 2 - 3 times per week (3)<br>❍ 4 - 5 times per week (4)<br>❍ 6 - 7 times per week (5)<br>❍ Don't now (998)<br>❍ Don't want to answer (999)	❍ Less than 30 minutes<br>❍ 30 - 59 minutes<br>❍ 60 - 119 minutes<br>❍ 2 - 4 hours<br>❍ More than 4 hours<br>❍ Don't now (998)<br>❍ Don't want to answer (999)
Team ball games, for example football, basketball, volley ball or floor ball *(Q730_7 PHY3_SPORTS_BAL_FRQ) (Q731_7 PHY3_SPORTS_BAL_DUR)*	❍ 1 - 3 times per month (1)<br>❍ 1 time per week (2)<br>❍ 2 - 3 times per week (3)<br>❍ 4 - 5 times per week (4)<br>❍ 6 - 7 times per week (5)<br>❍ Don't now (998)<br>❍ Don't want to answer (999)	❍ Less than 30 minutes<br>❍ 30 - 59 minutes<br>❍ 60 - 119 minutes<br>❍ 2 - 4 hours<br>❍ More than 4 hours<br>❍ Don't now (998)<br>❍ Don't want to answer (999)
Golf *(Q730_8 PHY3_SPORTS_GOL_FRQ) (Q731_8 PHY3_SPORTS_GOL_DUR)*	❍ 1 - 3 times per month (1)<br>❍ 1 time per week (2)<br>❍ 2 - 3 times per week (3)<br>❍ 4 - 5 times per week (4)<br>❍ 6 - 7 times per week (5)<br>❍ Don't now (998)<br>❍ Don't want to answer (999)	❍ Less than 30 minutes<br>❍ 30 - 59 minutes<br>❍ 60 - 119 minutes<br>❍ 2 - 4 hours<br>❍ More than 4 hours<br>❍ Don't now (998)<br>❍ Don't want to answer (999)
Dance course or competitive dancing e g couple dancing, ballet, jazz or street *(Q730_9 PHY3_SPORTS_DAN_FRQ) (Q731_9 PHY3_SPORTS_DAN_DUR)*	❍ 1 - 3 times per month (1)<br>❍ 1 time per week (2)<br>❍ 2 - 3 times per week (3)<br>❍ 4 - 5 times per week (4)<br>❍ 6 - 7 times per week (5)<br>❍ Don't now (998)<br>❍ Don't want to answer (999)	❍ Less than 30 minutes<br>❍ 30 - 59 minutes<br>❍ 60 - 119 minutes<br>❍ 2 - 4 hours<br>❍ More than 4 hours<br>❍ Don't now (998)<br>❍ Don't want to answer (999)
Horseback riding *(Q730_10 PHY3_SPORTS_HOR_FRQ) (Q731_10 PHY3_SPORTS_HOR_DUR)*	❍ 1 - 3 times per month (1)<br>❍ 1 time per week (2)<br>❍ 2 - 3 times per week (3)<br>❍ 4 - 5 times per week (4)<br>❍ 6 - 7 times per week (5)<br>❍ Don't now (998)<br>❍ Don't want to answer	❍ Less than 30 minutes<br>❍ 30 - 59 minutes<br>❍ 60 - 119 minutes<br>❍ 2 - 4 hours<br>❍ More than 4 hours<br>❍ Don't now (998)<br>❍ Don't want to answer (999)

	Number of times	Time per session
	(999)	
Ice-skating, ice hockey or bandy *(Q730_11 PHY3_SPORTS_HOC_FRQ) (Q731_11 PHY3_SPORTS_HOR_DUR)*	❍ 1 - 3 times per month (1)<br>❍ 1 time per week (2)<br>❍ 2 - 3 times per week (3)<br>❍ 4 - 5 times per week (4)<br>❍ 6 - 7 times per week (5)<br>❍ Don't now (998)<br>❍ Don't want to answer (999)	❍ Less than 30 minutes<br>❍ 30 - 59 minutes<br>❍ 60 - 119 minutes<br>❍ 2 - 4 hours<br>❍ More than 4 hours<br>❍ Don't now (998)<br>❍ Don't want to answer (999)
Skiing, downhill or cross country *(Q730_12 PHY3_SPORTS_SKI_FRQ) (Q731_12 PHY3_SPORTS_SKI_DUR)*	❍ 1 - 3 times per month (1)<br>❍ 1 time per week (2)<br>❍ 2 - 3 times per week (3)<br>❍ 4 - 5 times per week (4)<br>❍ 6 - 7 times per week (5)<br>❍ Don't now (998)<br>❍ Don't want to answer (999)	❍ Less than 30 minutes<br>❍ 30 - 59 minutes<br>❍ 60 - 119 minutes<br>❍ 2 - 4 hours<br>❍ More than 4 hours<br>❍ Don't now (998)<br>❍ Don't want to answer (999)
Martial Arts e g judo or karate *(Q730_13 PHY3_SPORTS_MAR_FRQ) (Q731_13 PHY3_SPORTS_MAR_DUR)*	❍ 1 - 3 times per month (1)<br>❍ 1 time per week (2)<br>❍ 2 - 3 times per week (3)<br>❍ 4 - 5 times per week (4)<br>❍ 6 - 7 times per week (5)<br>❍ Don't now (998)<br>❍ Don't want to answer (999)	❍ Less than 30 minutes<br>❍ 30 - 59 minutes<br>❍ 60 - 119 minutes<br>❍ 2 - 4 hours<br>❍ More than 4 hours<br>❍ Don't now (998)<br>❍ Don't want to answer (999)
Yoga, Pilates or Tai chi *(Q730_15 PHY3_SPORTS_YOG_FRQ) (Q731_15 PHY3_SPORTS_YOG_DUR)*	❍ 1 - 3 times per month (1)<br>❍ 1 time per week (2)<br>❍ 2 - 3 times per week (3)<br>❍ 4 - 5 times per week (4)<br>❍ 6 - 7 times per week (5)<br>❍ Don't now (998)<br>❍ Don't want to answer (999)	❍ Less than 30 minutes<br>❍ 30 - 59 minutes<br>❍ 60 - 119 minutes<br>❍ 2 - 4 hours<br>❍ More than 4 hours<br>❍ Don't now (998)<br>❍ Don't want to answer (999)
Boxing or wrestling *(Q730_14 PHY3_SPORTS_BOX_FRQ) (Q731_14 PHY3_SPORTS_BOX_DUR)*	❍ 1 - 3 times per month (1)<br>❍ 1 time per week (2)<br>❍ 2 - 3 times per week (3)<br>❍ 4 - 5 times per week (4)<br>❍ 6 - 7 times per week (5)<br>❍ Don't now (998)<br>❍ Don't want to answer (999)	❍ Less than 30 minutes<br>❍ 30 - 59 minutes<br>❍ 60 - 119 minutes<br>❍ 2 - 4 hours<br>❍ More than 4 hours<br>❍ Don't now (998)<br>❍ Don't want to answer (999)
Tennis, badminton or table tennis *(Q730_20 PHY3_SPORTS_TEN_FRQ) (Q731_20 PHY3_SPORTS_TEN_DUR)*	❍ 1 - 3 times per month (1)<br>❍ 1 time per week (2)<br>❍ 2 - 3 times per week (3)<br>❍ 4 - 5 times per week (4)	❍ Less than 30 minutes<br>❍ 30 - 59 minutes<br>❍ 60 - 119 minutes<br>❍ 2 - 4 hours<br>❍ More than 4 hours

	Number of times	Time per session
	❍ 6 - 7 times per week (5)<br>❍ Don't now (998)<br>❍ Don't want to answer (999)	❍ Don't now (998)<br>❍ Don't want to answer (999)
Squash *(Q730_21 PHY3_SPORTS_SQU_FRQ) (Q731_21 PHY3_SPORTS_SQU_DUR)*	❍ 1 - 3 times per month (1)<br>❍ 1 time per week (2)<br>❍ 2 - 3 times per week (3)<br>❍ 4 - 5 times per week (4)<br>❍ 6 - 7 times per week (5)<br>❍ Don't now (998)<br>❍ Don't want to answer (999)	❍ Less than 30 minutes<br>❍ 30 - 59 minutes<br>❍ 60 - 119 minutes<br>❍ 2 - 4 hours<br>❍ More than 4 hours<br>❍ Don't now (998)<br>❍ Don't want to answer (999)
Sailing, surfing, canoe or row *(Q730_17 PHY3_SPORTS_SAI_FRQ) (Q731_17 PHY3_SPORTS_SAI_DUR)*	❍ 1 - 3 times per month (1)<br>❍ 1 time per week (2)<br>❍ 2 - 3 times per week (3)<br>❍ 4 - 5 times per week (4)<br>❍ 6 - 7 times per week (5)<br>❍ Don't now (998)<br>❍ Don't want to answer (999)	❍ Less than 30 minutes<br>❍ 30 - 59 minutes<br>❍ 60 - 119 minutes<br>❍ 2 - 4 hours<br>❍ Morer than 4 hours<br>❍ Don't now (998)<br>❍ Don't want to answer (999)
Motorsport e g motocross *(Q730_18 PHY3_SPORTS_MOT_FRQ) (Q731_18 PHY3_SPORTS_MOT_DUR)*	❍ 1 - 3 times per month (1)<br>❍ 1 time per week (2)<br>❍ 2 - 3 times per week (3)<br>❍ 4 - 5 times per week (4)<br>❍ 6 - 7 times per week (5)<br>❍ Don't now (998)<br>❍ Don't want to answer (999)	❍ Less than 30 minutes<br>❍ 30 - 59 minutes<br>❍ 60 - 119 minutes<br>❍ 2 - 4 hours<br>❍ More than 4 hours<br>❍ Don't now (998)<br>❍ Don't want to answer (999)
Climbing *(Q730_19 PHY3_SPORTS_CLI_FRQ) (Q731_19 PHY3_SPORTS_CLI_DUR)*	❍ 1 - 3 times per month (1)<br>❍ 1 time per week (2)<br>❍ 2 - 3 times per week (3)<br>❍ 4 - 5 times per week (4)<br>❍ 6 - 7 times per week (5)<br>❍ Don't now (998)<br>❍ Don't want to answer (999)	❍ Less than 30 minutes<br>❍ 30 - 59 minutes<br>❍ 60 - 119 minutes<br>❍ 2 - 4 hours<br>❍ More than 4 hours<br>❍ Don't now (998)<br>❍ Don't want to answer (999)

q784 *(PHY3_PROF)*

Do you participate in sport competitions regularly?

❍ Yes (1) [Exclusive]
❍ No (0) [Exclusive]
❍ Don't know (998) [Keep position ◆ Exclusive]
❍ Don't want to answer(999) [Keep position ◆ Exclusive]

Dietary habits

The following section concerns dietary habits.

q1041

di2. How often do you eat or drink at the following occasions?

Breakfast *(Q1041_1 DIE_MEALS_BREA_FRQ)*	❍ Every day [1]<br>❍ Several times per week [2]<br>❍ Sometime every week [3]<br>❍ More seldom or never [4]<br>❍ Don't know [998]<br>❍ Don't want to answer [999]
Lunch *(Q1041_3 DIE_MEALS_LUNC_FRQ)*	❍ Every day [1]<br>❍ Several times per week [2]<br>❍ Sometime every week [3]<br>❍ More seldom or never [4]<br>❍ Don't know [998]<br>❍ Don't want to answer [999]
Dinner/supper *(Q1041_5 DIE_MEALS_DINN_FRQ)*	❍ Every day [1]<br>❍ Several times per week [2]<br>❍ Sometime every week [3]<br>❍ More seldom or never [4]<br>❍ Don't know [998]<br>❍ Don't want to answer [999]

q1274 *(DIE_MEALS_COFF_FRQ)*

di3. How often do you take snack meals?

❍ 4 times or more per day [1] =4
❍ 3 times per day [2] =3
❍ 1-2 times per day [3] =2
❍ Sometime every week [4] =1
❍ More seldom or never [5] =0
❍ Don't know [998]
❍ Don't want to answer [999]

Beverages

g1184

di4. Regarding the beverages you drink **at least once a month**, choose from the drop down menu below *how often* you usually drink these.

Only fill out those that you usually drink.

	Times per day	Times per week
Water (tap or bottle), 1 glass *(Q1181_1 DIE2_BEVERAGE_WATE_DAY) (Q1182_1 DIE2_BEVERAGE_WATE_WEEK)*	❍ 1 [1]<br>❍ 2 [2]<br>❍ 3 [3]<br>❍ 4 [4]<br>❍ 5+ [5]	❍ 1-2 [1]<br>❍ 3-4 [2]<br>❍ 5-6 [3]
Coffee *(Q1181_2 DIE2_BEVERAGE_COFF_DAY) (Q1182_2 DIE2_BEVERAGE_COFF_WEEK*	❍ 1 [1]<br>❍ 2 [2]<br>❍ 3 [3]<br>❍ 4 [4]<br>❍ 5+ [5]	❍ 1-2 [1]<br>❍ 3-4 [2]<br>❍ 5-6 [3]
Tea *(Q1181_3 DIE2_BEVERAGE_TEA_DAY) (Q1182_3 DIE2_BEVERAGE_TEA_WEEK)*	❍ 1 [1]<br>❍ 2 [2]<br>❍ 3 [3]<br>❍ 4 [4]<br>❍ 5+ [5]	❍ 1-2 [1]<br>❍ 3-4 [2]<br>❍ 5-6 [3]
Milk, 1 glass or bowl *(Q1181_4 DIE2_BEVERAGE_MILK_DAY) (Q1182_4 DIE2_BEVERAGE_MILK_WEEK)*	❍ 1 [1]<br>❍ 2 [2]<br>❍ 3 [3]<br>❍ 4 [4]<br>❍ 5+ [5]	❍ 1-2 [1]<br>❍ 3-4 [2]<br>❍ 5-6 [3]
Juice *(Q1181_5 DIE2_BEVERAGE_JUIC_DAY) (Q1182_5 DIE2_BEVERAGE_JUIC_WEEK)*	❍ 1 [1]<br>❍ 2 [2]<br>❍ 3 [3]<br>❍ 4 [4]<br>❍ 5+ [5]	❍ 1-2 [1]<br>❍ 3-4 [2]<br>❍ 5-6 [3]
Soda, cider, table drink or lemonade *(Q1181_6 DIE2_BEVERAGE_SODA_DAY) (Q1182_6 DIE2_BEVERAGE_SODA_WEEK)*	❍ 1 [1]<br>❍ 2 [2]<br>❍ 3 [3]<br>❍ 4 [4]<br>❍ 5+ [5]	❍ 1-2 [1]<br>❍ 3-4 [2]<br>❍ 5-6 [3]

q947 Coffee

You have stated that you drink coffee.

di4a. From the following alternatives, mark what you usually put in your coffee:

❑ Milk or cream (e.g. in cappuccino, latte) [1] *(Q947_1 DIE_BEVERAGE_COFF_MILK)*
❑ Sugar or syrup [2] *(Q947_2 DIE_BEVERAGE_COFF_SUGA)*
❑ Sweetener [3] *(Q947_3 DIE_BEVERAGE_COFF_SWEE)*
❍ None of the above [4] *(Q947_4 DIE_BEVERAGE_COFF_0)*
❍ Don't know [998] *(Q947_998 DIE_BEVERAGE_COFF_998)*
❍ Don't want to answer [999] *(Q947_999 DIE_BEVERAGE_COFF_999)*

q1168 Tea

You have stated that you drink tea.

di4b. Mark what sort/sorts of tea you usually drink

❑ Black tea [1] *(Q1168_1 DIE_BEVERAGE_TEA_BLAC)*
❑ Green tea [2] *(Q1168_2 DIE_BEVERAGE_TEA_GREE)*
❑ Red tea [3] *(Q1168_3 DIE_BEVERAGE_TEA_RED)*
❑ Herbal tea [4] *(Q1168_4 DIE_BEVERAGE_TEA_HERB)*
❑ Other [101] *(Q1168_5 DIE_BEVERAGE_TEA_OTH)*
❍ Don't know [998] *(Q1168_998 DIE_BEVERAGE_TEA_998)*
❍ Don't want to answer [999] *(Q1168_999 DIE_BEVERAGE_TEA_999)*

Food - Bread, sour milk, cereals, porridge

g1085

di5. Regarding the type of food you eat **at least once a month**, choose from the drop down menu below *how often* you eat it.

Only fill out those that you usually eat.

	Times per day	Times per week
White bread (e.g. tin loaf, loaf or flatbread [1] *(Q789_1 DIE2_FOOD1A_BREW_DAY) (Q1102_1 DIE2_FOOD1A_BREW_WEEK)*	❍ 1 [1]<br>❍ 2 [2]<br>❍ 3 [3]<br>❍ 4 [4]<br>❍ 5+ [5]	❍ 1-2 [1]<br>❍ 3-4 [2]<br>❍ 5-6 [3]
Soft brown bread (e.g. rye bread, whole meal bread, Rusk) [2] *(Q789_2 DIE2_FOOD1A_BRED_DAY)(Q1102_2 DIE2_FOOD1A_BRED_WEEK)*	❍ 1 [1]<br>❍ 2 [2]<br>❍ 3 [3]<br>❍ 4 [4]<br>❍ 5+ [5]	❍ 1-2 [1]<br>❍ 3-4 [2]<br>❍ 5-6 [3]
Crisp bread [3] *(Q789_3 DIE2_FOOD1A_BRER_DAY9) (Q1102_3 DIE2_FOOD1A_BRER_WEEK)*	❍ 1 [1]<br>❍ 2 [2]<br>❍ 3 [3]<br>❍ 4 [4]<br>❍ 5+ [5]	❍ 1-2 [1]<br>❍ 3-4 [2]<br>❍ 5-6 [3]
Processed sour milk, yoghurt or yoghurt drink [4] *(Q789_4 DIE2_FOOD1A_YOGH_DAY) (Q1102_4 DIE2_FOOD1A_YOGH_WEEK)*	❍ 1 [1]<br>❍ 2 [2]<br>❍ 3 [3]<br>❍ 4 [4]	❍ 1-2 [1]<br>❍ 3-4 [2]<br>❍ 5-6 [3]

	Times per day	Times per week
	❍ 5+ [5]	
Muesli or cereals [5] *(Q789_5 DIE2_FOOD1A_MUSL_DAY) (Q1102_5 DIE2_FOOD1A_MUSL_WEEK)*	❍ 1 [1]<br>❍ 2 [2]<br>❍ 3 [3]<br>❍ 4 [4]<br>❍ 5+ [5]	❍ 1-2 [1]<br>❍ 3-4 [2]<br>❍ 5-6 [3]
Oatmeal porridge [6] *(Q789_6 DIE2_FOOD1A_OAT_DAY) (Q1102_6 DIE2_FOOD1A_OAT_WEEK)*	❍ 1 [1]<br>❍ 2 [2]<br>❍ 3 [3]<br>❍ 4 [4]<br>❍ 5+ [5]	❍ 1-2 [1]<br>❍ 3-4 [2]<br>❍ 5-6 [3]

q1116 Bread slices *(DIE2_FOOD1A_BREAD_AMOUNT)*

You have stated that you eat bread.

di5a. How many slices of bread do you usually eat at each time?

❍ 1-2 slices [1]
❍ 3-4 slices [2]
❍ 5-6 slices [3]
❍ 7 slices or more [4]
❍ Don't know [998]
❍ Don't want to answer [999]

q1169 Fat on bread

di5b. What kind of butter/margarine do you usually use on your bread? Mark one or several alternatives.

❑ Bregott [1] *(Q1169_1 DIE2_FOOD1A_BREAD_BREG)*
❑ Margarine (e.g. Lätta, Lätt & Lagom, Milda lätt, Becel) [2] *(Q1169_2 DIE2_FOOD1A_BREAD_MARL)*
❑ Cholesterol-lowering margarine (e.g. Becel proactiv) [3] *(Q1169_3 DIE2_FOOD1A_BREAD_COLE)*
❑ Butter [4] *(Q1169_4 DIE2_FOOD1A_BREAD_BUTT)*
❑ Other [101] *(Q1169_101 DIE2_FOOD1A_BREAD_OTH)*
❍ None [0] *(Q1169_0 DIE2_FOOD1A_BREAD_NO)*
❍ Don't know [998] *(Q1169_998 DIE2_FOOD1A_BREAD_BREG)*
❍ Don't want to answer [999] *(Q1169_999 DIE2_FOOD1A_BREAD_BREG)*

q1207 Sour milk/yoghurt

You have stated that you eat processed sour milk/yoghurt.

di5c. Mark which one/ones of the following sorts of processed sour milk/yoghurts you usually eat?

❑ With added bacterial culture (natural or with flavor, e.g. A-fil, Verum hälsofil, Onaka, Actimel) [1] *(Q1207_1 DIE2_FOOD1A_YOGHURT_BACT)*
❑ Natural [2] *(Q1207_2 DIE2_FOOD1A_YOGHURT_NATU)*
❑ With fruit-, berries- or vanilla flavor [3] *(Q1207_3 DIE2_FOOD1A_YOGHURT_FRUI)*
❑ Other [101] *(Q1207_101 DIE2_FOOD1A_YOGHURT_OTH)*
❍ Don't know [998] *(Q1207_998 DIE2_FOOD1A_YOGHURT_998)*
❍ Don't want to answer [999] *(Q1207_999 DIE2_FOOD1A_YOGHURT_999)*

q1209 Cereals

You have stated that you eat muesli/cereals.

di5d. Mark which one/ones of the following sorts of muesli or cereals you usually eat?

❑ Corn flakes or Special K [1] *(Q1209_1 DIE2_FOOD1A_MUSLI_CORN)*
❑ Start, Crunchy or sweetened cereals (e.g. Frosties, Kalaspuffar) [2] *(Q1209_2 DIE2_FOOD1A_MUSLI_STAR)*
❑ Muesli or wholegrain cereals (e.g. All Bran) [3] *(Q1209_3 DIE2_FOOD1A_MUSLI_MUSL)*
❑ Other [101] *(Q1209_101 DIE2_FOOD1A_MUSLI_OTH)*
❍ Don't know [998] *(Q1209_998 DIE2_FOOD1A_MUSLI_998)*
❍ Don't want to answer [999] *(Q1209_999 DIE2_FOOD1A_MUSLI_999)*

Food – Cheese, sandwich meat, egg, flaxseeds

g1278

di6. Regarding the type of food you eat **at least once a month**, choose from the drop down menu how often you eat it.

Only fill out those that you usually eat.

	Times per day	Times per week
Cheese [1] *(Q1276_1 DIE2_FOOD1B_CHE_DAY)*	❍ 1 [1]<br>❍ 2 [2]	❍ 1-2 [1]<br>❍ 3-4 [2]

	Times per day	Times per week
(Q1277_1 DIE2_FOOD1B_CHE_WEEK)	❍ 3 [3]<br>❍ 4 [4]<br>❍ 5+ [5]	❍ 5-6 [3]
Cold cuts (e.g. ham, salami) [2] *(Q1276_2 DIE2_FOOD1B_MEAT_DAY) (Q1277_2 DIE2_FOOD1B_MEAT_WEEK)*	❍ 1 [1]<br>❍ 2 [2]<br>❍ 3 [3]<br>❍ 4 [4]<br>❍ 5+ [5]	❍ 1-2 [1]<br>❍ 3-4 [2]<br>❍ 5-6 [3]
Egg or omelet [3] *(Q1276_3 DIE2_FOOD1B_EGG_DAY) (Q1277_3 DIE2_FOOD1B_EGG_WEEK)*	❍ 1 [1]<br>❍ 2 [2]<br>❍ 3 [3]<br>❍ 4 [4]<br>❍ 5+ [5]	❍ 1-2 [1]<br>❍ 3-4 [2]<br>❍ 5-6 [3]
Flaxseed [4] *(Q1276_4 DIE2_FOOD1B_LINS_DAY) (Q1277_4 DIE2_FOOD1B_LINS_WEEK)*	❍ 1 [1]<br>❍ 2 [2]<br>❍ 3 [3]<br>❍ 4 [4]<br>❍ 5+ [5]	❍ 1-2 [1]<br>❍ 3-4 [2]<br>❍ 5-6 [3]

q1171 Cheese *(DIE2_FOOD1B_CHEEZE)*

You stated that you eat cheese.

di6a. How many slices of cheese do you usually eat at each occasion?

❍ 1-2 slices [1]
❍ 3-4 slices [2]
❍ 5-6 slices [3]
❍ 7-8 slices [4]
❍ 9-10 slices [5]
❍ 11 slices or more [6]
❍ Don't know [998]
❍ Don't want to answer [999]

Food – Fruit

g1112

di7. Regarding the type of food you eat **at least once a month**, choose in the drop down menu how often you eat it.

Only fill out those that you usually eat.

	Times per week	Times per month
Banana [1]*(Q1111_1 DIE2_FOOD2_BANA_WEEK) (Q1165_1 DIE2_FOOD2_BANA_MNT)*	❍ 1-2 [1]<br>❍ 3-4 [2]<br>❍ 5-6 [3]<br>❍ 7+ [4]	❍ 1-3 [1]
Apple or pear [2]*(Q1111_2 DIE2_FOOD2_APPL_WEEK) (Q1165_2 DIE2_FOOD2_APPL_MNT)*	❍ 1-2 [1]<br>❍ 3-4 [2]<br>❍ 5-6 [3]<br>❍ 7+ [4]	❍ 1-3 [1]
Orange, smaller citrus fruits or grapefruit [3] *(Q1111_3 DIE2_FOOD2_ORAN_WEEK)(Q1165_3 DIE2_FOOD2_ORAN_MNT)*	❍ 1-2 [1]<br>❍ 3-4 [2]<br>❍ 5-6 [3]<br>❍ 7+ [4]	❍ 1-3 [1]
Other fruits or berries [101] *(Q1111_101 DIE2_FOOD2_OTHF_WEEK) (Q1165_101 DIE2_FOOD2_OTHF_MNT)*	❍ 1-2 [1]<br>❍ 3-4 [2]<br>❍ 5-6 [3]<br>❍ 7+ [4]	❍ 1-3 [1]

Food – Cookies, chocolate, candy, ice cream, nuts, chips

g1284

di8. Regarding the type of food you eat **at least once a month**, choose in the drop down menu how often you eat it.

Only fill out those that you usually eat.

	Times per week	Times per month
Crackers, biscuits or cookies [1] *(Q1282_1 DIE2_FOOD3_BISC_WEEK) (Q1283_1 DIE2_FOOD3_BISC_MNT)*	❍ 1-2 [1]<br>❍ 3-4 [2]<br>❍ 5-6 [3]<br>❍ 7+ [4]	❍ 1-3 [1]
Bun, muffin or sponge cake [2] *(Q1282_2 DIE2_FOOD3_MUFF_WEEK) (Q1283_2 DIE2_FOOD3_MUFF_MNT)*	❍ 1-2 [1]<br>❍ 3-4 [2]<br>❍ 5-6 [3]<br>❍ 7+ [4]	❍ 1-3 [1]
Chocolate [3] *(Q1282_3 DIE2_FOOD3_CHOC_WEEK) (Q1283_3 DIE2_FOOD3_CHOC_MNT)*	❍ 1-2 [1]<br>❍ 3-4 [2]<br>❍ 5-6 [3]<br>❍ 7+ [4]	❍ 1-3 [1]

	Times per week	Times per month
Candy (not chocolate) [4] *(Q1282_4 DIE2_FOOD3_CAND_WEEK) (Q1283_4 DIE2_FOOD3_CAND_MNT)*	❍ 1-2 [1]<br>❍ 3-4 [2]<br>❍ 5-6 [3]<br>❍ 7+ [4]	❍ 1-3 [1]
Ice cream, sorbet or parfait [5] *(Q1282_5 DIE2_FOOD3_ICEC_WEEK) (Q1283_5 DIE2_FOOD3_ICEC_MNT)*	❍ 1-2 [1]<br>❍ 3-4 [2]<br>❍ 5-6 [3]<br>❍ 7+ [4]	❍ 1-3 [1]
Nuts, almonds or seeds (not flaxseed) [6] *(Q1282_6 DIE2_FOOD3_NUTS_WEEK) (Q1283_6 DIE2_FOOD3_NUTS_MNT)*	❍ 1-2 [1]<br>❍ 3-4 [2]<br>❍ 5-6 [3]<br>❍ 7+ [4]	❍ 1-3 [1]
Chips or cheese doodles [7] *(Q1282_7 DIE2_FOOD3_CHIP_WEEK) (Q1283_7 DIE2_FOOD3_CHIP_MNT)*	❍ 1-2 [1]<br>❍ 3-4 [2]<br>❍ 5-6 [3]<br>❍ 7+ [4]	❍ 1-3 [1]

q1194 Chocolate type

You stated that you eat chocolate.

di8a. Mark which or which ones of the following sorts of chocolate you usually eat?

❑ Pralines, Snickers, Daim, Japp or similar [1] *(Q1194_1 DIE2_FOOD3_CHOC_CREA)*
❑ Milk chocolate [2] *(Q1194_2 DIE2_FOOD3_CHOC_MILK)*
❑ Dark chocolate [3] *(Q1194_3 DIE2_FOOD3_CHOC_DARK)*
❑ White chocolate [4] *(Q1194_4 DIE2_FOOD3_CHOC_WHIT)*
❍ Don't know [998] *(Q1194_998 DIE2_FOOD3_CHOC_998)*
❍ Don't want to answer [999] *(Q1194_999 DIE2_FOOD3_CHOC_999)*

q1172 Chocolate amount *(DIE2_FOOD3_CHOC_AMOUNT)*

di8b. How much chocolate do you usually eat at each occasion? 1 large sized chocolate bar = 200 g.

1 Snickers or Daim = 50 g.

1 praline = 10 g.

❍ Less than 25 g [1]
❍ 25-50 g [2]
❍ 51-100 g [3]
❍ 101-200 g [4]
❍ More than 200 g [5]
❍ Don’t know [998]
❍ Don’t want to answer [999]

q1173 Candy amount *(DIE2_FOOD3_CANDY)*

You have stated that you eat candy.

D8c. How much candy do you usually eat at each occasion? Do not include chocolate.

1 bag of candy (e.g. “bilar” or “gott&blandat”) = 150 g.

Pastilles = 25 g.

❍ Less than 50 g [1]
❍ 50-99 g [2]
❍ 100-199 g [3]
❍ 200-299 g [4]
❍ 300 g or more [5]
❍ Don’t know [998]
❍ Don’t want to answer [999]

Cooked food

g753 Meat

di9. Regarding the type of meat dishes you eat **at least once a month**, choose from the drop down menu below how often you eat them.

Only fill out those that you usually eat.

	Times per week	Times per month

	Times per week	Times per month
Hamburger, kebab or tacos [1] *(Q751_1 DIE2_MEAL1_HAMB_WEEK) (Q752_1 DIE2_MEAL1_HAMB_MNT)*	❍ 1-2 [1]<br>❍ 3-4 [2]<br>❍ 5-6 [3]<br>❍ 7+ [4]	❍ 1-3 [1]
Ground beef dishes (e.g. bolognese, lasagna, meatballs, ground beef steak) [2] *(Q751_2 DIE2_MEAL1_MINC_WEEK) (Q752_2 DIE2_MEAL1_MINC_MNT)*	❍ 1-2 [1]<br>❍ 3-4 [2]<br>❍ 5-6 [3]<br>❍ 7+ [4]	❍ 1-3 [1]
Chicken or other poultry (e.g. roasted, cooked, stew, fried) [3] *(Q751_3 DIE2_MEAL1_CHIC_WEEK) (Q752_3 DIE2_MEAL1_CHIC_MNT)*	❍ 1-2 [1]<br>❍ 3-4 [2]<br>❍ 5-6 [3]<br>❍ 7+ [4]	❍ 1-3 [1]
Sausage (e.g. roasted, cooked, stew, gratin) [4] *(Q751_4 DIE2_MEAL1_SAUS_WEEK) (Q752_4 DIE2_MEAL1_SAUS_MNT)*	❍ 1-2 [1]<br>❍ 3-4 [2]<br>❍ 5-6 [3]<br>❍ 7+ [4]	❍ 1-3 [1]
Pork or beef (e.g. chop, filet, stew) [5] *(Q751_5 DIE2_MEAL1_BEEF_WEEK) (Q752_5 DIE2_MEAL1_BEEF_MNT)*	❍ 1-2 [1]<br>❍ 3-4 [2]<br>❍ 5-6 [3]<br>❍ 7+ [4]	❍ 1-3 [1]
Lamb or game [6] *(Q751_6 DIE2_MEAL1_LAMB_WEEK) (Q752_6 DIE2_MEAL1_LAMB_MNT)*	❍ 1-2 [1]<br>❍ 3-4 [2]<br>❍ 5-6 [3]<br>❍ 7+ [4]	❍ 1-3 [1]
Black pudding or blood sausage [7] *(Q751_7 DIE2_MEAL1_BLAC_WEEK) (Q752_7 DIE2_MEAL1_BLAC_MNT)*	❍ 1-2 [1]<br>❍ 3-4 [2]<br>❍ 5-6 [3]<br>❍ 7+ [4]	❍ 1-3 [1]

g756 Fish

di10. Regarding the type of dishes you eat **at least once a month**, choose from the drop down menu below how often you eat them.

Only fill out those that you usually eat.

	Times per week	Times per month
White fish (e.g. cod, pollock, fish fingers, fish balls) [1] *(Q754_1 DIE2_MEAL2_FISH_WEEK) (Q755_1 DIE2_MEAL2_FISH_MNT)*	❍ 1-2 [1]<br>❍ 3-4 [2]<br>❍ 5-6 [3]<br>❍ 7+ [4]	❍ 1-3 [1]
Salmon, sushi, herring or mackerel [2] *(Q754_2 DIE2_MEAL2_SALM_WEEK) (Q755_2 DIE2_MEAL2_SALM_MNT)*	❍ 1-2 [1]<br>❍ 3-4 [2]<br>❍ 5-6 [3]<br>❍ 7+ [4]	❍ 1-3 [1]
Tuna [3] *(Q754_3 DIE2_MEAL2_TUNA_WEEK) (Q755_3 DIE2_MEAL2_TUNA_MNT)*	❍ 1-2 [1]<br>❍ 3-4 [2]<br>❍ 5-6 [3]<br>❍ 7+ [4]	❍ 1-3 [1]

g903 Vegetarian dishes, soup, pie

di11. Regarding the type of dishes you eat **at least once a month**, choose from the drop down menu below how often you eat them.

Only fill out those that you usually eat.

	Times per week	Times per month
Vegetarian courses (e.g. lentil stew, bean, soy sausage, quorn) [4] *(Q901_4 DIE2_MEAL2_VEGO_WEEK) (Q902_4 DIE2_MEAL2_VEGO_MNT)*	❍ 1-2 [1]<br>❍ 3-4 [2]<br>❍ 5-6 [3]<br>❍ 7+ [4]	❍ 1-3 [1]
Salad courses [5] *(Q901_5 DIE2_MEAL2_SALL_WEEK) (Q902_5 DIE2_MEAL2_SALL_MNT)*	❍ 1-2 [1]<br>❍ 3-4 [2]<br>❍ 5-6 [3]<br>❍ 7+ [4]	❍ 1-3 [1]
Baguette with filling, sandwich or wrap etc [6] *(Q901_6 DIE2_MEAL2_BAGU_WEEK) (Q902_6 DIE2_MEAL2_BAGU_MNT)*	❍ 1-2 [1]<br>❍ 3-4 [2]<br>❍ 5-6 [3]<br>❍ 7+ [4]	❍ 1-3 [1]
Soup [7] *(Q901_7 DIE2_MEAL2_SOUP_WEEK) (Q902_7 DIE2_MEAL2_SOUP_MNT)*	❍ 1-2 [1]<br>❍ 3-4 [2]<br>❍ 5-6 [3]<br>❍ 7+ [4]	❍ 1-3 [1]
Pizza, pie or pasty [8] *(Q901_8 DIE2_MEAL2_PIZZ_WEEK) (Q902_8*	❍ 1-2 [1]<br>❍ 3-4 [2]<br>❍ 5-6 [3]	❍ 1-3 [1]

DIE2_MEAL2_PIZZ_MNT)	❍ 7+ [4]	
Pancakes, small pancakes, batter pudding, waffles [9] (Q901_9 DIE2_MEAL2_PANN_WEEK) (Q902_9 DIE2_MEAL2_PANN_MNT)	❍ 1-2 [1]<br>❍ 3-4 [2]<br>❍ 5-6 [3]<br>❍ 7+ [4]	❍ 1-3 [1]

q771 Vegetarian dishes

You have stated that you eat vegetarian courses.

di11a. Mark the courses you usually eat.

❑ Bean-, lentil- or chick pea courses (e.g. lentil stew, falafel) [1] *(Q771_1 DIE2_MEAL2_VEGO_BEAN)*
❑ Soy beef, soy sausage or tofu [2] *(Q771_2 DIE2_MEAL2_VEGO_SOYA)*
❑ Quorn or quorn stew [3] *(Q771_3 DIE2_MEAL2_VEGO_QUOR)*
❑ Root vegetable courses [4] *(Q771_4 DIE2_MEAL2_VEGO_ROOT)*
❍ None [0] *(Q771_0 DIE2_MEAL2_VEGO_NO)*
❍ Don't know [998] *(Q771_998 DIE2_MEAL2_VEGO_998)*
❍ Don't want to answer [999] *(Q771_999 DIE2_MEAL2_VEGO_999)*

q773 Salad courses

You have stated that you eat salad courses.

di11b. Mark the ingredients that your salad dish usually contains.

❑ Pasta, couscous, bulgur or quinoa [1] *(Q773_1 DIE2_MEAL2_SALLAD_PAST)*
❑ Cheese (e.g. hard cheese, feta cheese, mozzarella or cottage cheese) [2] *(Q773_2 DIE2_MEAL2_SALLAD_CHEE)*
❑ Ham, sausage, salami or roast beef [3] *(Q773_3 DIE2_MEAL2_SALLAD_HAM)*
❑ Chicken or turkey [4] *(Q773_4 DIE2_MEAL2_SALLAD_CHIC)*
❑ Salmon, tuna or shellfish [5] *(Q773_5 DIE2_MEAL2_SALLAD_SALM)*
❑ Beans, lentils or peas [6] *(Q773_6 DIE2_MEAL2_SALLAD_BEAN)*
❑ Other [101] *(Q773_101 DIE2_MEAL2_SALLAD_OTH)*
❍ None [0] *(Q773_0 DIE2_MEAL2_SALLAD_NO)*
❍ Don't know [998] *(Q773_998 DIE2_MEAL2_SALLAD_998)*
❍ Don't want to answer [999] *(Q773_999 DIE2_MEAL2_SALLAD_999)*

q775 Baguette

You have stated that you eat baguettes with filling, sandwiches, wraps etc.

di11c. Mark what kind of ingredients this type of food usually consists of.

❑ Cheese (e.g. hard cheese, feta cheese, mozzarella or cottage cheese) [1] *(Q775_1 DIE2_MEAL2_BAGUETTE_CHEE)*
❑ Ham, sausage, salami, meatballs or roast beef [2] *(Q775_2 DIE2_MEAL2_BAGUETTE_HAM)*
❑ Chicken or turkey [3] *(Q775_3 DIE2_MEAL2_BAGUETTE_CHIC)*
❑ Salmon, tuna or shellfish [4] *(Q775_4 DIE2_MEAL2_BAGUETTE_SALM)*
❑ Vegetables [5] *(Q775_5 DIE2_MEAL2_BAGUETTE_VEGE)*
❑ Fillings (e.g. shrimp, tuna, beetroot) [6] *(Q775_6 DIE2_MEAL2_BAGUETTE_SKAG)*
❑ Other [101] *(Q775_101 DIE2_MEAL2_BAGUETTE_OTH)*
❑ None [0] *(Q775_0 DIE2_MEAL2_BAGUETTE_NO)*
❍ Don't know [998] *(Q775_998 DIE2_MEAL2_BAGUETTE_998)*
❍ Don't want to answer [999] *(Q775_999 DIE2_MEAL2_BAGUETTE_999)*

q776 Soup

You have stated that you eat soup.

di11d. Mark what kind of soups you usually eat.

❑ Readymade soups (e.g. Kelda, Blå Band, Campbells) [1] *(Q776_1 DIE2_MEAL2_SOUP_READ)*
❑ Fish and/or shellfish soup [2] *(Q776_2 DIE2_MEAL2_SOUP_FISH)*
❑ Meat and/or chicken soup [3] *(Q776_3 DIE2_MEAL2_SOUP_MEAT)*
❑ Pea soup [4] *(Q776_4 DIE2_MEAL2_SOUP_PEA)*
❑ Vegetable soup [5] *(Q776_5 DIE2_MEAL2_SOUP_VEGE)*
❑ Other [101] *(Q776_101 DIE2_MEAL2_SOUP_OTH)*
❍ None [0] *(Q776_0 DIE2_MEAL2_SOUP_NO)*
❍ Don't know [998] *(Q776_998 DIE2_MEAL2_SOUP_998)*
❍ Don't want to answer [999] *(Q776_999 DIE2_MEAL2_SOUP_999)*

Food - Potato, pasta, rice, vegetables, sauces and dressings

g759

di12. Regarding the kind of food you eat **at least once a month**, choose from the drop down menu how often you usually eat it.

Only fill out those that you usually eat.

	Times per week	Times per month
Boiled potatoes, mashed potatoes or baked potatoes [1] *(Q757_1 DIE2_FOOD4_POTA_WEEK) (Q758_1 DIE2_FOOD4_POTA_MNT)*	❍ 1-2 [1]<br>❍ 3-4 [2]<br>❍ 5-6 [3]<br>❍ 7+ [4]	❍ 1-3 [1]

	Times per week	Times per month
French fries, fried or wedges, hash of fried diced meat or potato gratin [2] *(Q757_2 DIE2_FOOD4_POMM_WEEK) (Q758_2 DIE2_FOOD4_POMM_MNT)*	❍ 1-2 [1]<br>❍ 3-4 [2]<br>❍ 5-6 [3]<br>❍ 7+ [4]	❍ 1-3 [1]
Pasta (3) *(Q757_3 DIE2_FOOD4_PAST_WEEK) (Q758_3 DIE2_FOOD4_PAST_MNT)*	❍ 1-2 [1]<br>❍ 3-4 [2]<br>❍ 5-6 [3]<br>❍ 7+ [4]	❍ 1-3 [1]
Rice [4] *(Q757_4 DIE2_FOOD4_RICW_WEEK) (Q758_4 DIE2_FOOD4_RICW_MNT)*	❍ 1-2 [1]<br>❍ 3-4 [2]<br>❍ 5-6 [3]<br>❍ 7+ [4]	❍ 1-3 [1]
Couscous, bulgur or quinoa [5] *(Q757_5 DIE2_FOOD4_COUS_WEEK) (Q758_5 DIE2_FOOD4_COUS_MNT)*	❍ 1-2 [1]<br>❍ 3-4 [2]<br>❍ 5-6 [3]<br>❍ 7+ [4]	❍ 1-3 [1]

g762 Vegetables

di13. Regarding the vegetables that you eat **at least once a month**, choose from the drop down menu how often you usually eat these.

Only fill out those that you usually eat.

	Times per week	Times per month
Mixed vegetables (e.g. wok) [1] *(Q760_1 DIE2_VEGE_MIXV_WEEK) (Q761_1 DIE2_VEGE_MIXV_MNT)*	❍ 1-2 [1]<br>❍ 3-4 [2]<br>❍ 5-6 [3]<br>❍ 7+ [4]	❍ 1-3 [1]
Tomatoes, cucumber or salad leaves [2] *(Q760_2 DIE2_VEGE_TOMA_WEEK) (Q761_2 DIE2_VEGE_TOMA_MNT)*	❍ 1-2 [1]<br>❍ 3-4 [2]<br>❍ 5-6 [3]<br>❍ 7+ [4]	❍ 1-3 [1]
Spinach or rocket salad [3] *(Q760_3 DIE2_VEGE_SPIN_WEEK) (Q761_3 DIE2_VEGE_SPIN_MNT)*	❍ 1-2 [1]<br>❍ 3-4 [2]<br>❍ 5-6 [3]<br>❍ 7+ [4]	❍ 1-3 [1]
Onion, leek or garlic [4] *(Q760_4 DIE2_VEGE_ONIO_WEEK) (Q761_4*	❍ 1-2 [1]<br>❍ 3-4 [2]<br>❍ 5-6 [3]	❍ 1-3 [1]

	Times per week	Times per month
DIE2_VEGE_ONIO_MNT)	❍ 7+ [4]	
Carrots [5] *(Q760_5 DIE2_VEGE_CARR_WEEK) (Q761_5 DIE2_VEGE_CARR_MNT)*	❍ 1-2 [1]<br>❍ 3-4 [2]<br>❍ 5-6 [3]<br>❍ 7+ [4]	❍ 1-3 [1]
Broccoli, Brussels sprouts or Chinese cabbage [6] *(Q760_6 DIE2_VEGE_BRUS_WEEK) (Q761_6 DIE2_VEGE_BRUS_MNT)*	❍ 1-2 [1]<br>❍ 3-4 [2]<br>❍ 5-6 [3]<br>❍ 7+ [4]	❍ 1-3 [1]
Avocado [7] *(Q760_7 DIE2_VEGE_AVOC_WEEK) (Q761_7 DIE2_VEGE_AVOC_MNT)*	❍ 1-2 [1]<br>❍ 3-4 [2]<br>❍ 5-6 [3]<br>❍ 7+ [4]	❍ 1-3 [1]
Olives [8] *(Q760_8 DIE2_VEGE_OLIV_WEEK) (Q761_8 DIE2_VEGE_OLIV_MNT)*	❍ 1-2 [1]<br>❍ 3-4 [2]<br>❍ 5-6 [3]<br>❍ 7+ [4]	❍ 1-3 [1]

g765 Sauces and dressing

di14. Regarding the sauces and dressings you eat **at least once a month**, choose from the drop down menu below how often you usually eat them.

Only fill out those that you usually eat.

	Times per week	Times per month
Ketchup, tomato sauce, chili sauce or tomato salsa [1] *(Q763_1 DIE2_SAUC_TOMA_WEEK) (Q764_1 DIE2_SAUC_TOMA_MNT)*	❍ 1-2 [1]<br>❍ 3-4 [2]<br>❍ 5-6 [3]<br>❍ 7+ [4]	❍ 1-3 [1]
Vinaigrette (oil and vinegar) [2] *(Q763_2 DIE2_SAUC_VINA_WEEK) (Q764_2 DIE2_SAUC_VINA_MNT)*	❍ 1-2 [1]<br>❍ 3-4 [2]<br>❍ 5-6 [3]<br>❍ 7+ [4]	❍ 1-3 [1]
Cream sauce, sour cream or sour cream sauce [3] *(Q763_3 DIE2_SAUC_CREM_WEEK) (Q764_3*	❍ 1-2 [1]<br>❍ 3-4 [2]<br>❍ 5-6 [3]	❍ 1-3 [1]

	Times per week	Times per month
DIE2_SAUC_CREM_MNT)	❍ 7+ [4]	

Portion sizes (pictures not shown)

di15. Mark the picture that best matches the portions you usually eat.

q787

	[0]	[1]	[2]	[3]	[4]	[5]	Don’t know [998]	Don’t want to answer [999]
Potatoes, rice, pasta etc *(q787_1 DIE_PORT_POTA_1)*	❍	❍	❍	❍	❍	❍	❍	❍

q785

	[0]	[1]	[2]	[3]	[4]	[5]	Don’t know [998]	Don’t want to answer [999]
Meat, fish or vegetarian alternative *(q785_1 DIE_PORT_PROT_1)*	❍	❍	❍	❍	❍	❍	❍	❍

q788

	[0]	[1]	[2]	[3]	[4]	[5]	Don’t know [998]	Don’t want to answer [999]
Vegetables (raw or cooked) *(q788_1 DIE_PORT_VEGE_1)*	❍	❍	❍	❍	❍	❍	❍	❍

Fat, salt and light products

q777 Cooking fat

di16. Mark which edible fat/fats you usually use when cooking or baking.

❑ Butter [1] *(Q777_1 DIE_MISC_FAT_BUTT)*
❑ Margarine (e.g. Milda) [2] *(Q777_2 DIE_MISC_FAT_MARG)*
❑ Liquid margarine (e.g. Milda, Becel) [3] *(Q777_3 DIE_MISC_FAT_FLYT)*
❑ Liquid mix of butter- and rapeseed oil [4] *(Q777_4 DIE_MISC_FAT_MIXT)*
❑ Rapeseed oil [5] *(Q777_5 DIE_MISC_FAT_RAPS)*
❑ Olive oil [6] *(Q777_6 DIE_MISC_FAT_OLIV)*
❑ Cooking oil (e.g. corn-, sunflower oil) [7] *(Q777_7 DIE_MISC_FAT_OIL)*
❑ Other [101] *(Q777_101 DIE_MISC_FAT_OTH)*
❍ I don't use any cooking fat [0] *(Q777_0 DIE_MISC_FAT_NO)*
❍ Don't know [998] *(Q777_998 DIE_MISC_FAT_998)*
❍ Don't want to answer [999] *(Q777_999 DIE_MISC_FAT_999)*

q778 Salt

di17. Mark if any of the following statements matches your food habits.

❑ I usually use salt when I cook [1] *(Q778_1 DIE_MISC_SALT_COOK)*
❑ I usually salt my food on the plate [2] *(Q778_2 DIE_MISC_SALT_FOOD)*
❍ None of the above [0] *(Q778_0 DIE_MISC_SALT_NO)*
❍ Don't know [998] (Q778_998 DIE_MISC_SALT_998)
❍ Don't want to answer [999] (Q778_999 DIE_MISC_SALT_999)

q779 Light products *(DIE_MISC_LIGHT_FRQ)*

di18. Do you usually eat or drink sugar free food or low fat products?

❍ Yes, often [2]
❍ Yes, sometimes [1]
❍ Seldom or never [0]
❍ Don't know [998]
❍ Don't want to answer [999]

q780

di18a. Mark what kind of sugar free or low fat ("light products")food you normally choose.

❑ Soft drink, table drink, lemonade [1] *(Q780_1 DIE_MISC_LIGHT_SODA)*
❑ Jam, ice cream, candy, cookies [2] *(Q780_2 DIE_MISC_LIGHT_JAM)*
❑ Cheese [3] *(Q780_3 DIE_MISC_LIGHT_CHEE)*
❑ Milk, processed sour milk, yoghurt [4] *(Q780_4 DIE_MISC_LIGHT_MILK)*
❑ Other dairy products (e.g. cream, sour cream) [5] *(Q780_5 DIE_MISC_LIGHT_CREA)*
❑ Sauces and dressings [6] *(Q780_6 DIE_MISC_LIGHT_SAUC)*
❑ Meat products (e.g. sausage, salami, liver paste) [7] *(Q780_7 DIE_MISC_LIGHT_PREP)*
❑ Other [101] *(Q780_101 DIE_MISC_LIGHT_OTH)*
❑ *None of the above (Q780_0 DIE_MISC_LIGHT_NO)*
❍ Don't know [998] *(Q780_998 DIE_MISC_LIGHT_998)*
❍ Don't want to answer [999] *(Q780_999*

DIE_MISC_LIGHT_999)

Eating out

q781

di19. Mark the statement/statements below that best matches your eating habits.

❑ I have lunch in a fast food restaurant, food stall, pizzeria (incl. take away) at least 4 times per week [1] *(Q781_1 DIE2_HABITS_FAST)*
❑ I have lunch or dinner in a restaurant (incl. take away) at least 4 times per week [2] *(Q781_2 DIE2_HABITS_REST)*

❍ None of the above [0] *(Q781_3 DIE2_HABITS_NO)*
❍ Don't know [998] *(Q781_4 DIE2_HABITS_998)*
❍ Don't want to answer [999] *(Q781_5 DIE2_HABITS_999)*

q782 Salad buffet *(DIE2_RESTAURANT)*

di19a. In a restaurant, do you usually eat from the salad buffet?

❍ Yes, every week [3]
❍ Yes, a couple of times a month [2]
❍ Seldom or never [1]
❍ Don't know [998]
❍ Don't want to answer [999]

Food supplements

q769 *(Q769 DIE2_SUPPL_USE)*

di20. Do you usually take vitamins, minerals or other supplements?

❍ Yes, regularly [2]
❍ Yes, sometimes [1]
❍ No [0]
❍ Don't know [998]
❍ Don't want to answer [999]

g768

di20a. How often do you usually take the following dietary supplements? Fill out your intake during **the last months**.

Multivitamins or minerals [1]<br><br>*(Q766_1 DIE2_SUPPL_VIT_MULT_FRQ)*	❍ Every day [4]<br>❍ A couple of times a week [3]<br>❍ A couple of times a month [2]<br>❍ In periods [1]
Vitamin A [2]<br><br>*(Q766_2 DIE2_SUPPL_VIT_VITA_FRQ)*	❍ Every day [4]<br>❍ A couple of times a week [3]<br>❍ A couple of times a month [2]<br>❍ In periods [1]
Vitamin B (any kind) [3]<br><br>*(Q766_3 DIE2_SUPPL_VIT_VITB_FRQ)*	❍ Every day [4]<br>❍ A couple of times a week [3]<br>❍ A couple of times a month [2]<br>❍ In periods [1]
Vitamin C [4]<br><br>*(Q766_4 DIE2_SUPPL_VIT_VITC_FRQ)*	❍ Every day [4]<br>❍ A couple of times a week [3]<br>❍ A couple of times a month [2]<br>❍ In periods [1]
Vitamin D [5]<br><br>*(Q766_5 DIE2_SUPPL_VIT_VITD_FRQ)*	❍ Every day [4]<br>❍ A couple of times a week [3]<br>❍ A couple of times a month [2]<br>❍ In periods [1]
Vitamin E [6]<br><br>*(Q766_6 DIE2_SUPPL_VIT_VITE_FRQ)*	❍ Every day [4]<br>❍ A couple of times a week [3]<br>❍ A couple of times a month [2]<br>❍ In periods [1]
Folic acid [7]<br><br>*(Q766_7 DIE2_SUPPL_VIT_FOLI_FRQ)*	❍ Every day [4]<br>❍ A couple of times a week [3]<br>❍ A couple of times a month [2]<br>❍ In periods [1]
Iron [8]<br><br>*(Q766_8 DIE2_SUPPL_VIT_IRON_FRQ)*	❍ Every day [4]<br>❍ A couple of times a week [3]<br>❍ A couple of times a month [2]<br>❍ In periods [1]
Calcium [9]<br><br>*(Q766_9 DIE2_SUPPL_VIT_CALC_FRQ)*	❍ Every day [4]<br>❍ A couple of times a week [3]<br>❍ A couple of times a month [2]<br>❍ In periods [1]
Omega-3 (incl. fish oil) [10]<br><br>*(Q766_10 DIE2_SUPPL_VIT_OMEG_FRQ)*	❍ Every day [4]<br>❍ A couple of times a week [3]<br>❍ A couple of times a month [2]<br>❍ In periods [1]

q770

di20b. Mark what kind of supplement/supplements you have taken regularly during **the last months**.

❑ Beta carotene [1] *(Q770_1 DIE_SUPPL_BETA)*
❑ Selenium [3] *(Q770_2 DIE_SUPPL_SELE)*

❍ Antioxidants (e.g. Bio-Antioxidant, Antioxidant Plus) [7] *(Q770_7 DIE_SUPPL_ANTI)*
❍ Phytoestrogens (e.g. Menosoy, Femiform) [8] *(Q770_8 DIE_SUPPL_PHYT)*

❑ Zink [4] *(Q770_4 DIE_SUPPL_ZINK)*
❑ Magnesium [5] *(Q770_5 DIE_SUPPL_MAGN)*
❑ Q10 [6] *(Q770_6 DIE_SUPPL_Q10)*

❍ Other [101] *(Q770_101 DIE_SUPPL_OTH)*
❍ None of the above [0] *(Q770_0 DIE_SUPPL_NO)*
❍ Don't know [998] *(Q770_998 DIE_SUPPL_998)*
❍ Don't want to answer [999] *(Q770_999 DIE_SUPPL_999)*
