# Supplementary material for: Cohort profile: Studies of Work Environment and Disease Epidemiology-Infections (SWEDE-I), a prospective cohort on employed adults in Sweden
Source: PLoS One. 2019 May 15;14(5):e0217012. doi: 10.1371/journal.pone.0217012 (PMC6519895; doi:10.1371/journal.pone.0217012)
Supplement: S6 File — (PDF) [file pone.0217012.s006.pdf]

Sick report - Codebook

(SA2) Personal code number:

(TA4) Time when question was answered (SICK_REPORT_TIME)
State the date for when you fell ill (SICK_REPORT_DATE)

(SA4) Has there been less than 15 days since you fell ill?
(SICK_LESS_15DAYS)

- Yes (1)
- No (2)
- Don't know/Don't want to answer (3)

(SA5) Did you fall ill suddenly, i.e. did you fall ill within a few hours?
(SICK_SUDDEN_SICKNESS)

- Yes (1)
- No (2)
- Don't know/Don't want to answer (3)

(SA6) Have you felt feverish since you fell ill? (SICK_FEVER)

- Yes (1)
- No (2)
- Don't know/Don't want to answer (3)

(SA7) Did you have a temperature higher than 38 degrees? (SICK_FEVER_38)

- Yes (1)
- No (2)
- Don’t know/Don’t want to answer (3)

(SA8) Have you had coughs any time since you fell ill? (SICK_COUGH)

- Yes (1)
- No (2)
- Don’t know/Don’t want to answer (3)

(SA9) Have you had muscular pain or other bodily pain since you fell ill? (SICK_BODY_ACHEC)

- Yes (1)
- No (2)
- Don’t know/Don’t want to answer (3)

(SA10) Have you had a runny nose since you fell ill? (SICK_RUNNY_NOSE)

- Yes (1)
- No (2)
- Don’t know/Don’t want to answer (3)

(SA11) Have you had a sore throat since you fell ill? (SICK_SORE_THROAT)

- Yes (1)
- No (2)
- Don’t know/Don’t want to answer (3)

(SA12) Have you had a headache since you fell ill? (SICK_HEADACHE)

- Yes (1)
- No (2)
- Don’t know/Don’t want to answer (3)

(SA13) Have you had a nausea since you fell ill? (SICK_NAUSEA)

- Yes (1)
- No (2)
- Don’t know/Don’t want to answer (3)

(SA14) Have you vomited since you fell il? (SICK_VOMITING)

- Yes (1)
- No (2)
- Don’t know/Don’t want to answer (3)

(SA15) Have you had diarrhea any time since you fell ill? (SICK_DIARRHEA)

- Yes (1)
- No (2)
- Don’t know/Don’t want to answer (3)

(SA16) Have you felt breathless any time since you fell ill? (SICK_BREATHLESSNESS)

- Yes (1)
- No (2)
- Don’t know/Don’t want to answer (3)

(SA17) Have you visited the doctor any time since you fell ill? (SICK_VISIT_DOCTOR)

- Yes (1)
- No (2)
- Don’t know/Don’t want to answer (3)

(SA18) Did the doctor tell you it was an infection? (SICK_VISIT_DOCTOR_INFECTION)

- Yes (1)
- No (2)
- Don’t know/Don’t want to answer (3)

(SA19) Press the number that corresponds to the number of days since you noticed your illness. (SICK_DAYS)

Today (0)

1 (1)

2 (2)

3 (3)

4 (4)

5 (5)

6 (6)

7 (7)

>7 (8)

Don't know (9)

(SA20) During the week before you fell ill, did anyone in your family have symptoms similar to the ones you have now reported? (SICK_FAMILY)

- Yes (1)
- No (2)
- Don't know/Don't want to answer (3)

(SA21) Did anyone at your workplace have similar symptoms? (SICK_WORKPLACE)

- Yes (1)
- No (2)
- Don't know/Don't want to answer (3)

(SA22) Did anyone else in your environment have similar symptoms? (SICK_SURROUNDING)

- Yes (1)
- No (2)
- Don’t know/Don’t want to answer (3)

Thank you!
