# Supplementary material for: Cohort profile: Studies of Work Environment and Disease Epidemiology-Infections (SWEDE-I), a prospective cohort on employed adults in Sweden
Source: PLoS One. 2019 May 15;14(5):e0217012. doi: 10.1371/journal.pone.0217012 (PMC6519895; doi:10.1371/journal.pone.0217012)
Supplement: S1 Table — (DOCX) [file pone.0217012.s007.docx]

**S1 Table.** Comparison of baseline characteristics between cohort members and the working age Swedish population in 2011.

|  |  | SWEDE-I | Sweden 2011 |
| --- | --- | --- | --- |
|  | Age-class (years) | % | % |
|  |  |  |  |
| Age distribution^a^ | 25-34 | 13 | 24 |
|  | 35-44 | 32 | 26 |
|  | 45-54 | 26 | 26 |
|  | 55-64 | 29 | 24 |
|  |  |  |  |
| Gender^a^ (Men) | 25-64 | 41 | 51 |
|  |  |  |  |
| Smoking daily^b^ | 30-44 | 8 | 13 |
|  | 45-64 | 13 | 15 |
|  |  |  |  |
| Overweight and Obese^b^ | 30-44 | 47 | 47 |
|  | 45-64 | 57 | 59 |
|  |  |  |  |
| Elementary school^a^ (9 years or less) | 25-64 | 8 | 14 |
|  |  |  |  |
| Health status^b^ (Good and very good) | 30-44 | 81 | 81 |
|  | 45-64 | 80 | 70 |
| ^a^ Information on Swedish population available from Statistics Sweden | | |  |
| ^b^ Information on Swedish population available from Folkhälsomyndigheten, the Public Health Agency of Sweden | | | |
